# The draft genome of the C_3_ panicoid grass species *Dichanthelium oligosanthes*

**DOI:** 10.1186/s13059-016-1080-3

**Published:** 2016-10-28

**Authors:** Anthony J. Studer, James C. Schnable, Sarit Weissmann, Allison R. Kolbe, Michael R. McKain, Ying Shao, Asaph B. Cousins, Elizabeth A. Kellogg, Thomas P. Brutnell

**Affiliations:** 1 Donald Danforth Plant Science Center, St. Louis, MO 63132 USA; 2 Present address: Department of Crop Sciences, University of Illinois Urbana-Champaign, Urbana, IL 61801 USA; 3 Present address: Department of Agronomy and Horticulture, University of Nebraska-Lincoln, Lincoln, NE 68588 USA; 4 School of Biological Sciences, Washington State University, Pullman, WA 99164 USA; 5 St. Jude Children’s Research Hospital, Pediatric Cancer Genome Project, Memphis, TN USA

**Keywords:** *Dichanthelium oligosanthes*, PACMAD, Panicoid grass, Photosynthesis, Carbonic anhydrase

## Abstract

**Background:**

Comparisons between C_3_ and C_4_ grasses often utilize C_3_ species from the subfamilies Ehrhartoideae or Pooideae and C_4_ species from the subfamily Panicoideae, two clades that diverged over 50 million years ago. The divergence of the C_3_ panicoid grass *Dichanthelium oligosanthes* from the independent C_4_ lineages represented by *Setaria viridis* and *Sorghum bicolor* occurred approximately 15 million years ago, which is significantly more recent than members of the Bambusoideae, Ehrhartoideae, and Pooideae subfamilies. *D. oligosanthes* is ideally placed within the panicoid clade for comparative studies of C_3_ and C_4_ grasses.

**Results:**

We report the assembly of the nuclear and chloroplast genomes of *D. oligosanthes*, from high-throughput short read sequencing data and a comparative transcriptomics analysis of the developing leaf of *D. oligosanthes*, *S. viridis*, and *S. bicolor*. Physiological and anatomical characterizations verified that *D. oligosanthes* utilizes the C_3_ pathway for carbon fixation and lacks Kranz anatomy. Expression profiles of transcription factors along developing leaves of *D. oligosanthes* and *S. viridis* were compared with previously published data from *S. bicolor*, *Zea mays*, and *Oryza sativa* to identify a small suite of transcription factors that likely acquired functions specifically related to C_4_ photosynthesis.

**Conclusions:**

The phylogenetic location of *D. oligosanthes* makes it an ideal C_3_ plant for comparative analysis of C_4_ evolution in the panicoid grasses. This genome will not only provide a better C_3_ species for comparisons with C_4_ panicoid grasses, but also highlights the power of using high-throughput sequencing to address questions in evolutionary biology.

**Electronic supplementary material:**

The online version of this article (doi:10.1186/s13059-016-1080-3) contains supplementary material, which is available to authorized users.

## Background

The availability of complete genome sequences from multiple lineages is enabling a much deeper understanding of both the mechanistic basis of evolution and the diversification of gene regulatory networks. Furthermore, the breadth of genome sequences available provides opportunities to utilize non-model species in comparative genomics [1, 2]. Comparative approaches are made more powerful by sampling across the phylogenetic tree, particularly in cases of convergent evolution, and provide insight into the networks that underpin complex traits [3, 4]. High-throughput sequencing facilitates deep transcriptomic and genomic surveys, which can be leveraged to deduce the evolution of gene families by duplication and subsequent neofunctionalization and subfunctionalization of individual gene copies.

Growing concern over food and energy security has spurred translational research to increase the productivity and sustainability of crops. Optimization of photosynthesis is one approach that has the potential to greatly increase crop yields [5, 6]. Specifically, several groups are investigating the evolution of C_4_ from C_3_ photosynthesis with the objective of installing C_4_ traits into C_3_ species to improve yield [7]. Enhanced photosynthetic efficiency associated with C_4_ photosynthesis not only increases productivity (i.e. grain or biomass yield), but also nutrient and water use efficiency [8]. These benefits are the result of a carbon concentrating mechanism (CCM) that evolved to increases the CO_2_ concentration around the carboxylating enzyme ribulose-1,5-bisphosphate carboxylase/oxygenase (Rubisco). Concentrating CO_2_ around Rubisco reduces its oxygenase activity, thereby significantly decreasing the amount of energy lost to photorespiration. The CCM of the majority of C_4_ species is achieved through partitioning of the biochemical reactions of photosynthesis into two cell types (mesophyll, M, and bundle sheath, BS) [9].

Despite being a complex trait, C_4_ photosynthesis has independently evolved over 60 times in the angiosperms [10] and at least 22 times in the grasses [11]. The grasses are one of the most ecologically and economically significant plant clades and thus insights into the origins of C_4_ should provide opportunities for breeding and engineering improved germplasm. However, to date, comparative genomic approaches to studying the evolution of C_4_ photosynthesis have been limited to comparisons between crop species. These include C_4_ crops such as *Zea mays*, *Sorghum bicolor*, *Setaria italica*, and *Saccharum officinarum* from the clade containing the subfamilies Panicoideae, Arundinoideae, Chloridoideae, Micrairoideae, Aristidoideae, and Danthonioideae (PACMAD) and C_3_ crops such as *Oryza sativa* and *Triticum aestivum* from the clade containing the subfamilies Bambusoideae, Ehrhartoideae, and Pooideae (BEP). The limitations of PACMAD-BEP comparisons are that these two groups of grasses diverged more than 50 million years ago and the BEP clade contains no C_4_ species [11].

Distant evolutionary relationships sometimes fail to identify the genomic changes associated with the evolutionary emergence of C_4_ photosynthesis because differences in the photosynthetic pathway are confounded with the many other changes that occurred in the long independent history of the two lineages. The use of PACMAD-BEP comparisons has been driven by the availability of genomic resources. Currently the only published panicoid genome sequences are for panicoid species that utilize the C4 pathway for carbon fixation [12–15].

**Fig. 1:**
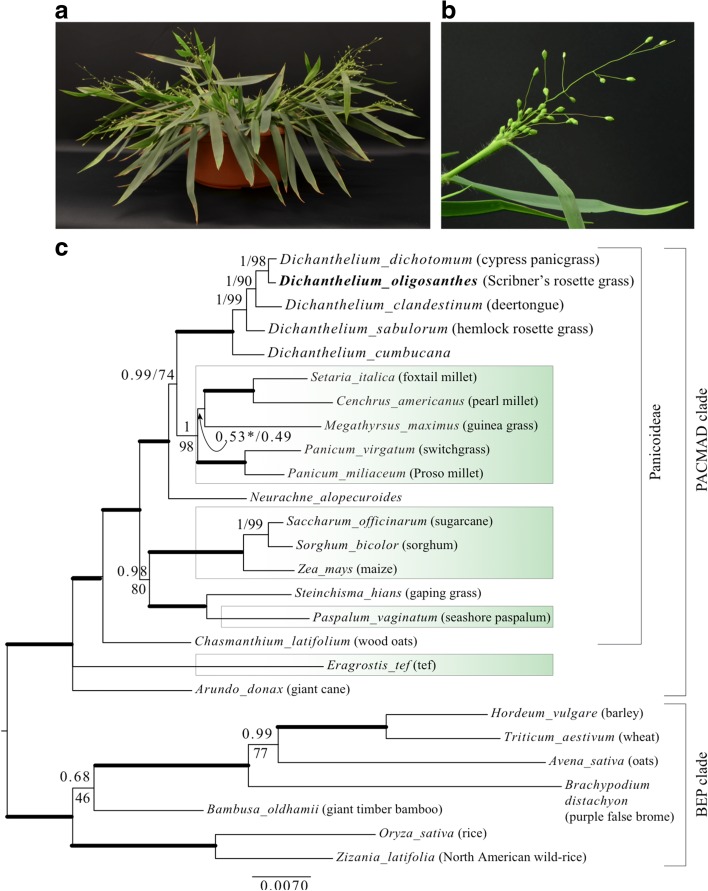
*D. oligosanthes* a C_3_ panicoid grass. A greenhouse grown *D. oligosanthes* plant (**a**), and panicle (**b**). **c** Bayesian tree of three chloroplast loci, showing the monophyly of *Dichanthelium* and placement of *D. oligosanthes*. Bayesian posterior probability values above branches, maximum likelihood bootstrap below; heavy branches received maximal support in both analyses (1.0, 100, respectively). The branch marked with * was resolved differently in the ML analysis but with low support. C_4_ clades are in *green shaded boxes*. The two major clades of grasses, PACMAD and BEP, are indicated by *brackets* as is subfamily Panicoideae

*Dichanthelium oligosanthes* is a C_3_ panicoid grass and thus an excellent species for comparisons to C_4_ panicoids such as *Z. mays*, *S. bicolor*, and *S. officinarum*, and species with an independent C_4_ origin represented by *S. italica*, *Cenchrus americanus*, *Panicum miliaceum*, and *Panicum virgatum* (see Fig. 1). Within the genus *Dichanthelium*, *D. oligosanthes* is reported to be diploid [16] and is widely distributed across North America (USDA, National Resource Conservation Service), increasing the accessibility of diverse germplasm and its utility in studying adaptation to abiotic stresses. Recently *D. clandestinum* was utilized in a comparative RNA sequencing (RNA-seq) experiment [17]. However, while transcriptomics data are useful, they do not provide key structural information (such as promoter and regulatory sequences) or evidence of orthology through syntenic relationships.

As the number of sequenced genomes increases, a more comprehensive understanding of the genes involved in C_4_ photosynthesis can be achieved. To this end, we sequenced and assembled a draft genome of *D. oligosanthes*. Histological, biochemical, and transcriptomic analyses confirm the C_3_ nature of *D. oligosanthes* and demonstrate its usefulness as a C_3_ panicoid grass for evolutionary comparisons. Furthermore, characteristics of *D. oligosanthes* also make it a potentially suitable genetic model for dissecting traits such as perenniality, cold tolerance, and flowering time. We demonstrate here how this high quality draft genome provides novel insights into the evolution and diversification of C_4_ photosynthesis in the grasses.

## Results and discussion

### Life history and phylogeny

The genus *Dichanthelium* includes ca. 72 species, which collectively are known as rosette grasses [18]. All species are perennial and plants overwinter as a rosette that grows to produce sparsely branched culms in the spring (Fig. 1) [18, 19] (AJS and EAK, personal observations). In many species the rosette leaves senesce late in the growing season, the culms develop more branches, and a second round of flowering occurs—hence the genus name, which means “twice-flowering.” Cleistogamy is common in *Dichanthelium* species [18]. While some of the inflorescences are borne well above the leaf sheath, others, particularly those from culm branches, never fully exert and self-pollinate without opening [18] (EAK, personal observations).

*Dichanthelium* is a member of the grass subfamily Panicoideae, tribe Paniceae. Like other members of Panicoideae, it has spikelets with two flowers, the upper one bisexual and the lower staminate or sterile (EAK, personal observations). Like other members of the tribe Paniceae, its chromosomes are in multiples of 9 [16]. Species of *Dichanthelium* are similar to but morphologically distinct from species of *Panicum*, so for many years *Dichanthelium* was treated as a subgenus of *Panicum* [20], a treatment that is still followed by some authors [21]. However, it was recognized as a distinct genus by Gould and Clark in 1978 [22].

Phylogenetic data support the distinction of *Dichanthelium* as a separate genus [11, 23, 24], showing that it is clearly not a lineage in the *Panicum* clade. Within the tribe Paniceae, the position of *Dichanthelium* is uncertain. Chloroplast sequences show that it is closely related to the large clade (the MPC clade) that includes groups of species utilizing different C_4_ photosynthesis subtypes: *Melinidinae* (PEPCK), *Panicinae* (NAD-ME), and *Cenchrinae* (NADP-ME) [25]. Depending on the sample of taxa and chloroplast sequences, *Dichanthelium* is either sister to the MPC plus the Australian species *Homopholis* and *Walwhalleya* [11] or as part of a larger clade including mostly C_3_ species but also the C_4_ members of the Australian Neurachninae [25]. In contrast, data from a single nuclear gene sequence place it sister to all Paniceae except *Echinochloa* [26]. In either case, it is more closely related to the C_4_ panicoid species than any of the C_3_ grasses for which complete genome assemblies are currently available (e.g. *O. sativa* and *B. distachyon*). The close relationship between *Dichanthelium* and C_4_ Paniceae is confirmed when sequences from the *D. oligosanthes* chloroplast genes *rbcL*, *ndhF*, and *matK* were used in maximum likelihood and Bayesian analyses with select species previously used to construct a phylogeny of the grasses (Fig. 1c) [11].

### Anatomy and physiology of *Dichanthelium oligosanthes*

**Fig. 2:**
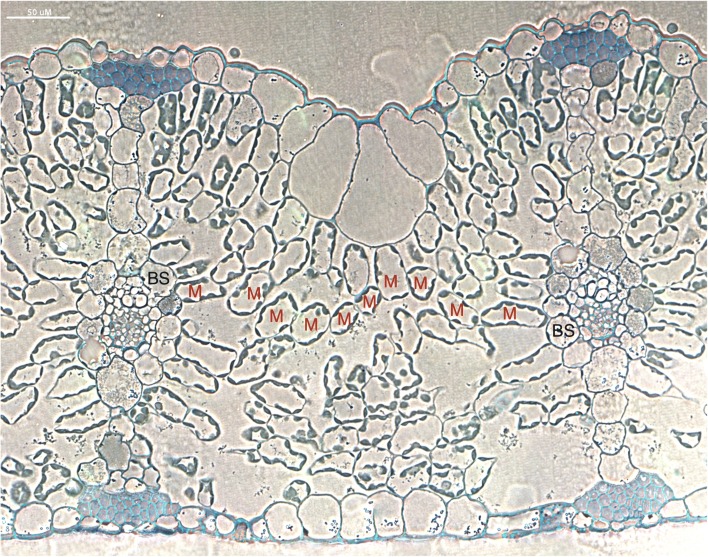
*D. oligosanthes* leaf *cross-section*. Toluidine blue-stained leaf cross-section shows stained chloroplasts present in mesophyll cells (M) but generally lacking in bundle sheath cells (BS). Wide vein spacing is apparent in the leaf, which is characteristic of a C_3_ species

Histological analysis of *D. oligosanthes* leaf cross-sections indicates that its anatomy is consistent with that of a temperate C_3_ grass. Vacuoles occupy the majority of the cell volume in M cells, with chloroplasts arranged near the cell periphery (Fig. 2). Large air spaces are present between loosely arranged spongy mesophyll cells. The BS cells of *D. oligosanthes* have few and small chloroplasts, a hallmark of C_3_ species (*S. bicolor* and *O. sativa* cross-sections can be found in [27]). The altered cellular arrangement, known as Kranz anatomy, that facilitates the CCM is clearly absent from the *D. oligosanthes* leaf cross-section. While many variations of Kranz anatomy have been identified [28–31], generally C_4_ species have narrow vein spacing, with large BS cells arranged around the vasculature and M cells surrounding the BS cells [32, 33]. In C_4_ Panicoideae species, typically two M cells separate consecutive BS (BS-M-M-BS), but *D. oligosanthes* has many mesophyll cells between veins, which is consistent with C_3_ leaf anatomy (Fig. 2).

**Fig. 3:**
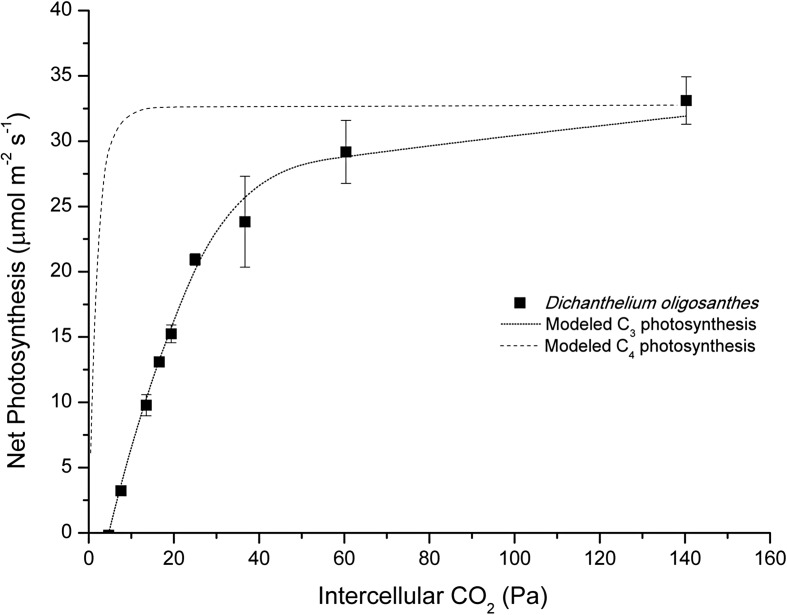
*D. oligosanthes* leaf gas exchange. Net CO_2_ assimilation in response to changes in intercellular CO_2_. *Squares* correspond to gas exchange data from *D. oligosanthes. Lines* represent modeled A–C_i_ curves in C_3_ and C_4_ photosynthesis

The CO_2_ response curve generated from *D. oligosanthes* is also typical of a C_3_ species (Fig. 3). The data fit well with the C_3_ model of photosynthesis, characterized by a higher compensation point (approximately 48.2 μbar) and a more gradual increase in photosynthesis at low *p*CO_2_ [34]. The initial slope of the response curve is typical of C_3_ photosynthesis whereas a much steeper slope is observed in C_4_ species.

Biochemical assays revealed typical activity levels of Rubisco, phosphoenolpyruvate carboxylase (PEPC), and carbonic anhydrase (CA) for a C_3_ monocot species (Table 1). The measured in vitro Rubisco activity corresponds well with V_cmax_ values estimated from gas exchange and is consistent with other C_3_ species. PEPC activity is low, as predicted by RNA-seq data and as expected for a C_3_ species where it likely functions in an anaplerotic role for TCA cycle activities. CA activity is within the broad range of CA activity levels in C_3_ grass species [35]. The plants measured here show high variation in total Rubisco and CA activity likely due to differences in plant maturity; however, all measured values are within the accepted ranges for C_3_ species [35–37].

**Table 1:** *D. oligosanthes* physiology and biochemical characteristics

Assay	Measurement	Published C_3_ values	Published C_4_ values
δ^13^C (‰)	–28.35 ± 0.37	–28.1 ± 2.5 [38]	–13.5 ± 1.5 [38]
Rubisco (μmol m^*−*2^ s^*−*1^)	31.3 ± 8.1	20–70 [36]	14–54 [36, 80]
PEPC (μmol m^*−*2^ s^*−*1^)	7.2 ± 1.9	2–6 [36, 37]	110–220 [36, 67, 80]
CA (μmol m^*−*2^ s^*−*1^)	901.4 ± 220.9	14–1673 [35]	2–1200 [35, 67, 71]

*D. oligosanthes* has a distinctively C_3_ isotopic signature (Table 1; [38]), which is consistent with other *Dichanthelium* species that have been previously reported [17, 39, 40]. This value reflects strong isotopic discrimination by Rubisco, demonstrating that CO_2_ fixation occurs via the C_3_ cycle. Both C_4_ species and Type II C_3_-C_4_ intermediates have distinct isotopic signatures indicating CO_2_ fixed by PEPC. However, type I intermediates have a C_3_-like isotopic signature, but can be differentiated by anatomical and biochemical characteristics that are more similar to C_4_ species [9, 41]. Taken together, the leaf anatomy, gas exchange, and biochemical measurements corroborate previous reports that *D. oligosanthes* is a C_3_ species [42].

### Nuclear and chloroplast genome assembly and annotation

To estimate the genome size of *D. oligosanthes*, flow cytometry and k-mer abundance assays were performed. Flow cytometry of *D. oligosanthes* accession *Kellogg 1175* produced an estimated genome size of approximately 960 Mb, placing it within the range for diploid panicoid grasses. Single copy sequences present a distinctive peak in histograms of k-mer abundance centered at the average depth of sequencing. Sequences repeated twice in the genome form a second peak at twice the average sequencing depth and so on. Based on k-mer analysis, the estimated genome size of *D. oligosanthes* was revised downward to 750 Mb of which approximately 360 Mb is single copy sequence.

Sequence analysis was performed on a single individual derived from self-pollination of a wild-collected individual (*Kellogg 1175*), collected at the Shaw Nature Reserve in Gray Summit, MO. *D. oligosanthes* is a predominately self-pollinating species, so heterozygosity was expected to be low. A *D. oligosanthes* draft assembly was generated using data from libraries with median 180 bp insert and 5 kb insert sizes. Sequencing was performed on an Illumina HiSeq 2000 platform with 100 bp paired end sequencing. Approximately 90 Gb and 86 Gb of sequence was generated from the 180 bp and 5 kb libraries, respectively. These data were assembled using Allpaths-LG [43]. Additional scaffolding was conducted using two mate pair insert libraries (5 kb median and 6.3 kb median insert size) and the software package SSPACE [44] and sequence present in a number of gaps was determined using GapCloster [45]. The final assembly consisted of 17,441 scaffolds (589 megabases), which were constructed from 76,905 contigs (476 megabases of sequence). The assembly we present here therefore covers 78 % of the estimated total genome of *D. oligosanthes*, including determined sequence for 63 % of the genome. Based on the alignment of a set of low copy genes conserved in *S. bicolor*, *S. italica*, and *Panicum hallii*, we determined that our assembly contains at least 98 % of the *D. oligosanthes* gene space (3358/3430 genes identified). A total of 30,153 genes were annotated through a combination of homology-based and de novo annotation using Maker2 [46]. For these genes, 1 kb of promoter sequence was recovered 94.2 % of the time, and 5 kb of promoter sequence was recovered 80.5 % of the time. 86.5 % of all annotated genes were present on multi-gene scaffolds, enabling syntenic comparisons to other grass genomes.

**Fig. 4:**
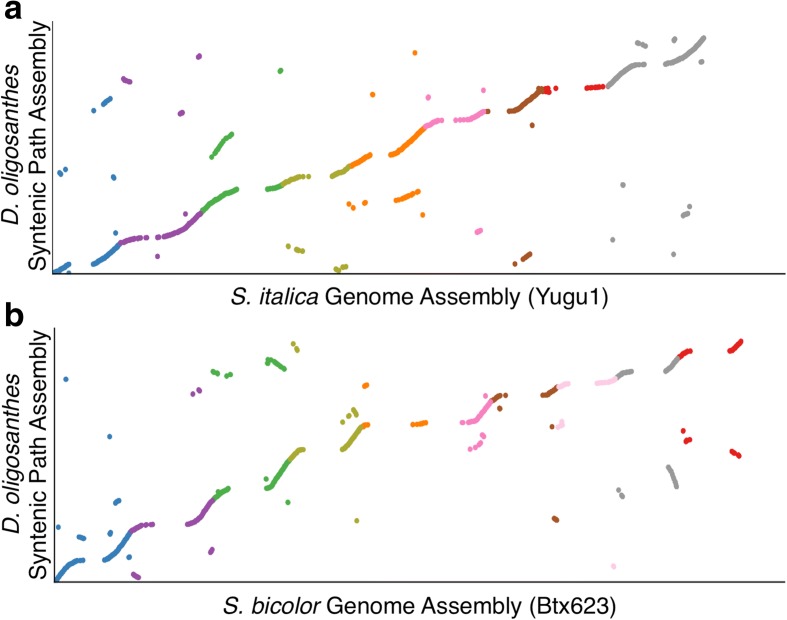
*D. oligosanthes* genome synteny with *S. italica* and *S. bicolor*. Syntenic path assemblies comparing the content of the *Dichanthelium oligosanthes* draft genome assembly to the two most closely related species with pseudomolecule level assemblies. **a** Comparison to *S. italica*. **b** Comparison to *S. bicolor*

The resulting assemblies and gene model annotations were loaded into CoGe to explore synteny relationships and enable community access to the datasets through iPlant servers [47, 48]. Through the use of CoGe’s syntenic path assembly algorithm [49], the *D. oligosanthes* scaffolds were compared to the genomes of *S. bicolor* and *S. italica* to show the overall coverage of the genome (Fig. 4). Coverage of the euchromatic arms is quite good, with most of the *D. oligosanthes* genome present in large enough scaffolds to be ordered and oriented based on syntenic data from close relatives. Centromeric and pericentromeric regions are not well represented in the syntenic path assemblies. This may be because pericentromeric regions are highly repetitive and are more difficult to assemble, and/or because pericentromeric regions tend to have lower gene content and fewer conserved genes between species. No evidence of significant gene-loss was identified when comparing syntenic orthologous regions of these three genomes, which confirms that this reference accession of *D. oligosanthes*, like *S. bicolor* and *S. italica*, and unlike *Z. mays*, is diploid relative to the common ancestor of the grasses.

In addition to the nuclear genome assembly, a subset of the data was used to create a de novo assembly of the chloroplast genome. An initial assembly was made using SPAdes v.3.1.0 [50] and the resulting contigs were further assembled using Sequencher (Genecodes— 5.2.4). Gaps between contigs were filled using raw sequencing reads to extend the contig ends (see “Methods”). The overlapping regions of the contigs were then verified by mapping reads to the junction in Sequencher. Read depth across the assembled chloroplast sequence was checked by estimating 20-mer abundance using Jellyfish v.2.1.3 [51] and mapping abundance to 20-mers across the assembly. No breaks or large shifts in coverage, other than what is expected at inverted repeat boundaries, were identified (Table 2). Annotation of the chloroplast genome was performed using Dual Organellar GenoMe Annotator (DOGMA) [52] and visualized with Circos [53] (see Additional file 1: Figure S1). No large-scale rearrangements or duplications were identified in the plastid genome of *D. oligosanthes* relative to other panicoid grass plastomes.

**Table 2:** *D. oligosanthes* chloroplast genome assembly statistics

	Total size	LSC	SSC	IR
Chloroplast genome	140,100	82,090	12,572	22,719

### Comparative analysis of genes expression across leaf development

One of the unique features of monocot leaves is that developmental processes proceed linearly, with the base segments being the least and the tip being the most differentiated [54]. This continuous gradient has been exploited previously to investigate the expression of genes related to photosynthesis in *Z. mays* and *O. sativa* [55] and *S. bicolor* and *S. viridis* [56]. As a C_3_ panicoid grass, *D. oligosanthes* provides a unique opportunity to examine the diversification of genes and networks associated with C_4_ photosynthesis using comparative transcriptomic approaches. To expand the gradient analyses, developmental leaf gradients were constructed for *D. oligosanthes* and the closely related C_4_ species *S. viridis*. The *S. viridis* gradient from [56] was not used because the data were not replicated.

**Fig. 5:**
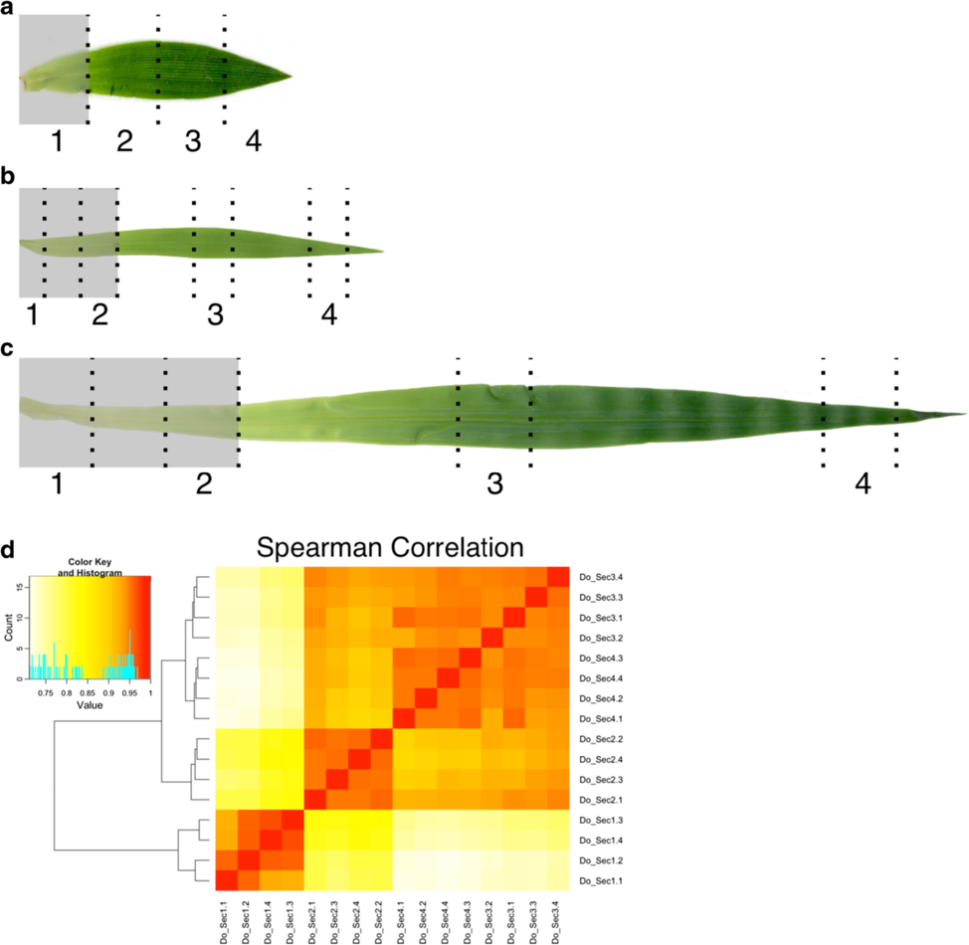
Leaf developmental gradients. The third leaf of **a** *D. oligosanthes*, **b** *S. viridis*, and **c** *S. bicolor. Shaded boxes* indicate the portion of the leaf shaded by the second leaf ligule. *Dotted lines* indicate the segments sampled for the expression analyses. **d** *Heatmap* showing a Spearman correlation of the *D. oligosanthes* replicated leaf segment RNA-seq data

The same growth conditions employed in previously published grass leaf gradients were used for *D. oligosanthes* and *S. viridis* to reduce environmental variation [55, 56]. Leaf gradients were generated by collecting four segments from the third leaf from each species (Fig. 5a and b). While leaf length varies among species, the developmental programs that establish the anatomy and biochemistry for photosynthesis proceed in the same direction and segments of the third leaf above the point at which it is enveloped by the sheath of the second leaf are expected to act as source tissues while those below remain sink tissues. The *S. viridis* leaves were sampled similarly to a previously published four segment gradient of *Z. mays*, which captured the basal, transition, maturing, and mature zones of the leaf [54]. Because the *D. oligosanthes* third leaf is small, only a single segment could be collected below the second leaf ligule, whereas two segments were collected in *S. viridis*. Thus segment 1 of *D. oligosanthes* captures the equivalent of both base and transition (segments 1 and 2) of *S. viridis* (Fig. 5a and b). The *D. oligosanthes* leaf above the ligule was divided into three equal segments. Leaf segments 1, 3, 7, and 12 of *S. bicolor* were used from [56] in the analyses because they are most similar to the segments collected in *D. oligosanthes* and *S. viridis*.

Hierarchical clustering of global gene expression profiles using Spearman correlation values (see Additional file 1: Figure S2) indicates that the replicates of each segment are strongly correlated and each segment clusters separately (Fig. 5d). Pearson correlation analysis produced similar results. The strong correlation between segments 3 and 4 suggests that the tip of the leaf may be fully mature. These new leaf gradients provide an opportunity to investigate a variety of biological processes, including changes in gene regulation linked to the evolution of C_4_ photosynthesis.

### C_4_ carbon shuttle gene expression

**Fig. 6:**
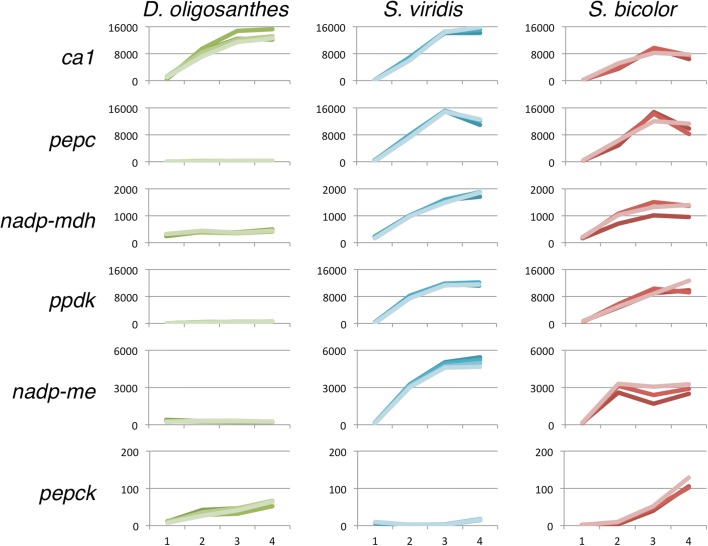
Expression profiles of core C_4_ enzymes. Expression profiles of six core C_4_ enzymes in *D. oligosanthes*, *S. viridis*, and *S. bicolor*. Expression values in FPKM are shown on the *y-axis* and the leaf segment is indicated on the *x-axis*. Transcriptional upregulation of these enzymes is not observed in *D. oligosanthes*

It is not surprising that all of the major enzymes involved in C_4_ photosynthesis are present in C_3_ species given that C_4_ photosynthesis has evolved from the C_3_ ancestral state over 70 times in the angiosperms [8]. Increased gene expression of the core C_4_ enzymes plays a major role in the evolution of C_4_ photosynthesis (reviewed in [9]). To investigate the primary method of carbon fixation for *D. oligosanthes*, we compared the expression of six core C_4_ enzymes in *D. oligosanthes* to its C_4_ relatives *S. viridis* and *S. bicolor* (Fig. 6, Additional file 2: Table S1).

Consistent with the results of the anatomical and physiological analyses, the expression profile of all six core C_4_ enzymes indicates that *D. oligosanthes* utilizes the ancestral C_3_ carbon fixation pathway. Large amounts of CA protein are known to be present in the leaves of C_3_ plants [57] and of the six genes encoding enzymes in the C_4_ pathway, only *carbonic anhydrase1* (*ca1*) was expressed at a high level in *D. oligosanthes* (Fig. 6). Significant accumulation of transcripts encoded by four other C_4_ genes was observed in *S. viridis* and *S. bicolor*. Expression levels of the C_4_ genes were similar in *S. bicolor* and *S. viridis* except for *ca1* and *nicotinamide adenine dinucleotide phosphate malic enzyme* (*nadp-me*), both of which are twofold lower in *S. bicolor* (Fig. 6). Although comparing absolute expression levels across species introduces numerous potential sources of bias and error, a difference in the number of tandemly duplicated *ca* gene copies likely explains the expression difference between *S. bicolor* and *S. viridis*. Unlike *ca*, *nadp-me* does not have highly expressed paralogs. Protein blot and enzyme activity assays would be needed to determine whether differences in enzymatic efficiency and/or differences in translational regulation compensate for the difference in transcriptional abundance for this gene.

Three major subtypes of C_4_ photosynthesis are recognized as (1) NADP-ME, (2) NAD-ME, and (3) PEPCK, named for the primary decarboxylating enzyme employed by each subtype. While traditionally these pathways have been viewed as independent, biochemical data and recent modeling of the C_4_ pathways revealed that the PEPCK pathway could be complementary in NADP-ME subtype species, such as *Z. mays*, *S. viridis*, and *S. bicolor* [58, 59]. Accordingly, although *Z. mays* is classified as an NADP-ME C_4_ subtype, the PEPCK pathway is likely active and contributes to total photosynthesis [60, 61]. This is reflected in the expression of *pepck1* in *Z. mays*, which accumulates to 2000 rpkm in the developing leaf tip and follows the expected expression profile of a gene involved in photosynthesis [55]. Interestingly, very low *pepck* expression levels were observed in both *S. viridis* and *S. bicolor* (Fig. 6). The reported lack of PEPCK protein in *S. bicolor* [62] confirms the expression result and provides further evidence that the PEPCK pathway is not active in these species. *Z. mays* and *S. bicolor* are believed to share a recent common ancestor [11], suggesting the acquisition of the PEPCK pathway in *Z. mays* may be a relatively new evolutionary innovation or, alternatively, that this secondary pathway was lost in *S. bicolor* after it diverged from *Z. mays*.

### C_4_ transcription factor identification

**Fig. 7:**
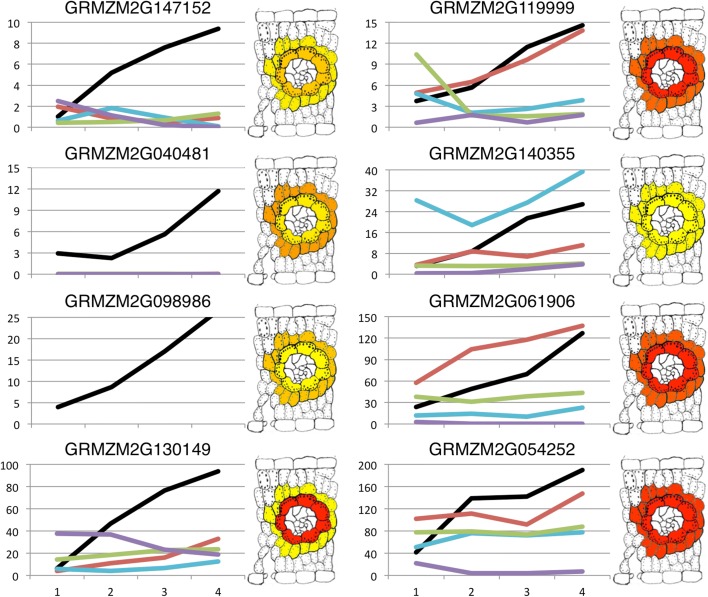
Putative C_4_ transcription factors. Expression profiles for each of the transcription factors identified in the comparative analysis. Expression values in FPKM are shown on the *y-axis* and the leaf segment is indicated on the *x-axis. Colored lines* represent each species as follows: *black*, *Z. mays*; *red*, *S. bicolor*; *blue*, *S. viridis*; *green*, *D. oligosanthes*; and *purple*, *O. sativa*. Leaf *cross-section diagrams* are *colored* to show the expression of each TF in M and BS cells of *Z. mays* [54]. Higher expression values are shown as *red*, while lower expression values are shown as *yellow*

The use of a single pairwise comparison between distantly related C_3_ and C_4_ species to identify genes linked to C_4_ photosynthesis is likely to produce large numbers of false positives. Any C_4_ panicoid differs from C_3_ BEP species in many aspects unrelated to the evolution of photosynthetic pathway. For example, a comparison of *Z. mays* and *O. sativa* leaves will identify gene expression differences that have accrued in the intervening 100 million years (50 million years on each branch since the common ancestor). Thus, there will have been fewer mutations between *Z. mays* and *D. oligosanthes* because the intervening time is 30 million years and fewer changes to be confounded with differences between C_3_ and C_4_. Multiple comparisons between species from two independent origins of C_4_ photosynthesis using a close C_3_ species for comparison can also distinguish changes that have occurred between the two C_4_ origins and are not directly related to C_4_. Changes that map to the *S. viridis* branch alone are specific to that lineage, whereas changes that appear independently on both the *S. viridis* and Andropogoneae branches are likely to reflect instances in which C_4_ has converged to use common genes. This would produce a shorter list of higher confidence gene candidates, ideally to the point where it would be practical to pursue functional validation of each individual gene. The leaf gradients of *D. oligosanthes*, *S. bicolor*, and *S. viridis* allow us to produce such a list. A previous study of a developmental leaf gradient in *Z. mays* identified three clusters of co-expressed genes correlated with photosynthetic activity [55]. These three clusters include 82 of the 1286 total transcription factors (TFs) annotated and expressed in the *Z. mays* leaf, 55 of which have an average expression across the leaf of at least 4 FPKM. Because general expression patterns were being compared across several species, this criterion was necessary for robust comparisons. Using the data from the leaf gradients reported here, an additional filter was imposed to restrict the list to only those TFs that showed a different expression profile in the leaves of *Z. mays*, *S. bicolor*, and/or *S. viridis* when compared to *D. oligosanthes* and *Oryza sativa*. Only eight TFs met these criteria (Fig. 7, Additional file 3: Table S2). Three of the eight TFs identified here are common to the 118 C_4_ TFs described by Wang et al. [55] (GRMZM2G130149, GRMZM2G061906, GRMZM2G119999), but none of these TFs were identified in a comparison between *Z. mays* and *Cleome gynandra* [63]. Four of the eight TFs displayed unique expression profiles in *Z. mays* (GRMZM2G147152, GRMZM2G040481, GRMZM2G098986, GRMZM2G130149). One of these (GRMZM2G098986) does not have an ortholog in the other species and is incorporated into a LTR transposable element. Three TFs in *Z. mays* share an expression profile with *S. bicolor* but not *S. viridis*, *D. oligosanthes*, or *Oryza sativa* (GRMZM2G119999, GRMZM2G061906, GRMZM2G054252). This result suggests that modified regulation of these TFs is specific to the Andropogoneae tribe rather than diagnostic of C_4_ function. A single TF shows a similar profile with all C_4_ panicoid species, but neither of the C_3_ species (GRMZM2G140355).

The four TFs identified that have expression profiles specific to *Z. mays* most likely result from the use of the *Z. mays* leaf developmental gradient as the initial filtering step and do not reflect a difference in the number of TFs with lineage-specific gene expression patterns among the species included in this analysis. However, given that the PEPCK pathway is specific to *Z. mays* in this small sample of C_4_ species, it is tempting to speculate that the *Z. mays* specific TFs contribute to regulation of the PEPCK pathway. Interestingly, utilizing data on cell type specific expression from the *Z. mays* eFP browser (http://bar.utoronto.ca/efp_maize/cgi-bin/efpWeb.cgi), a single TF was identified as preferentially expressed in bundle sheath cells, where PEPCK is needed for C_4_ photosynthesis. Taken together, these results suggest that the myb TF encoded by GRMZM2G130149 may be one of the genes that regulate the transcription of *pepck* in *Z. mays*.

### C_4_ specific amino acids under selection

In addition to probing the evolution of C_4_ photosynthesis using expression data, we also investigated amino acid substitutions in key C_4_ enzymes. Specific residues in several of the C_4_ carbon shuttle genes have been previously shown to be under positive selection in C_4_ lineages. These include PEPC [4], which encodes the first carboxylation reaction in C_4_ photosynthesis, the decarboxylating enzymes NADP-ME [64] and PEPCK [65], as well as the large subunit of Rubisco (rbcL) [66]. Peptide sequences for these genes in *D. oligosanthes* were compared to the known amino acid sequences across the grass family to identify signatures of selection in the amino acid sequences prior to the divergence of the C_4_ lineages from *D. oligosanthes*.

**Fig. 8:**
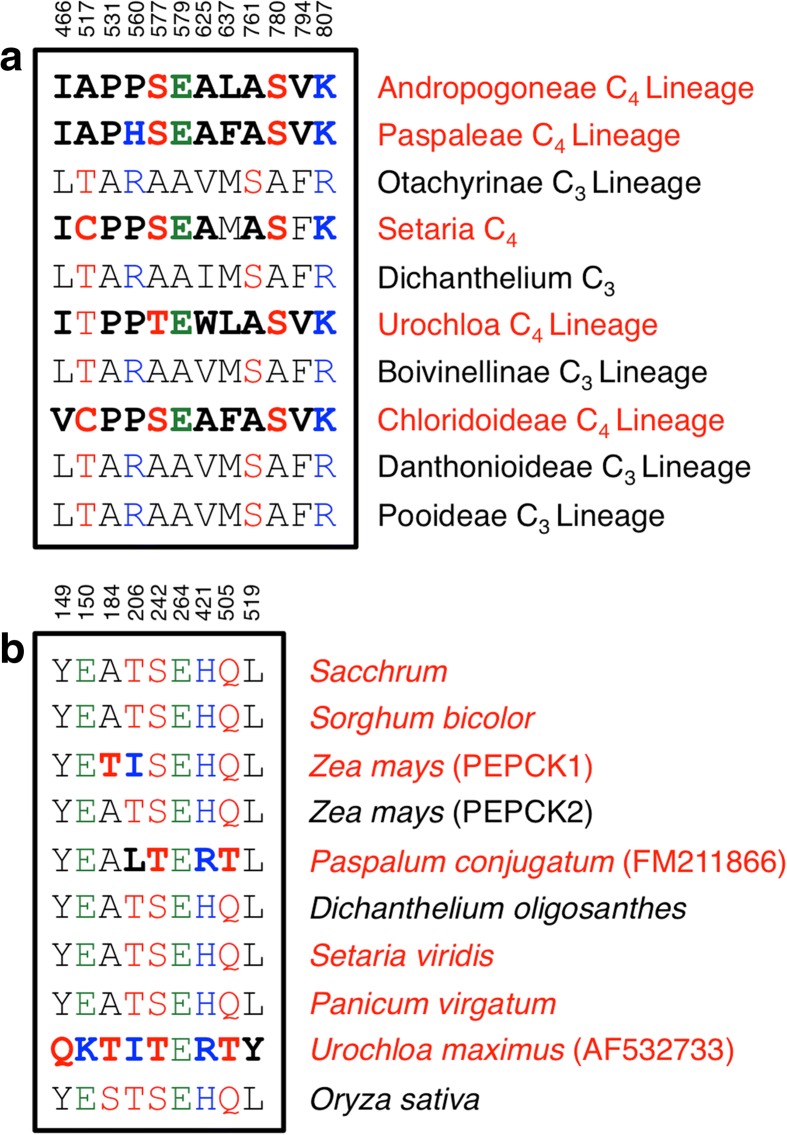
Amino acids under positive selection in C_4_ lineages. (**a**) PEPC and (**b**) PEPCK amino acid sequences for C_3_ and C_4_ grass species. Amino acids are *color-coded* according to their chemical properties: *black*, non-polar; *red*, polar; *green*, acidic; *blue*, basic. C_4_ amino acids are *bolded*. Figure modified from Christin et al. [4, 65]

Despite having sister taxa that evolved all three sub-types of C_4_ photosynthesis, *D. oligosanthes* does not contain the key amino acid substitutions present in the C_4_ enzymes. The most studied of these is the A780S substitution in PEPC. C_4_ lineages that have evolved the serine substitution are not inhibited by malate and require a lower PEP substrate concentration, which is advantageous for the C_4_, but not the C_3_ pathway [37]. While other amino acids in PEPC are under positive selection in C_4_ species, the A780S substitution is common to most C_4_ lineages and is absent in *D. oligosanthes* (Fig. 8a). A maximum-likelihood tree was used to ensure that the correct *D. oligosanthes* PEPC isoform was used for the analysis (see Additional file 1: Figure S3, Additional file 4: PEPC Amino Acid Sequences). Likewise, although most C_4_ species contain only a subset of the amino acids predominantly found among all C_4_ species, none of the prevailing C_4_ amino acid residues were present in *D. oligosanthes* for NADP-ME, PEPCK, or rbcL. Taken together, these results suggest that the common ancestor between *D. oligosanthes* and the closely related C_4_ species at the point of divergence was not under selective pressure for C_4_ photosynthesis or had not yet acquired mutations to develop C_4_. This also strongly supports the model that C_4_ photosynthesis was acquired independently in these diverged lineages and *D. oligosanthes* does not represent a loss or reversion to the C_3_ pathway [11].

Christin et al. identified a set of nine amino acid substitutions in the PEPCK gene that exhibited positive selection in species classified as the PEPCK C_4_ subtype, such as *Urochloa maximus* [65]. Of the two PEPCK genes present in *Z. mays*, the paralog that showed an expression pattern in our data consistent with a role in C_4_ photosynthesis (PEPCK1) contains two of the nine amino acid substitutions shown to be under positive selection in C_4_ species that utilize the PEPCK pathway. However, the copy that does not show a photosynthesis-linked pattern of expression (PEPCK2) does not contain any of the positively selected amino acid substitutions. In *S. bicolor* and *S. viridis*, where no expression evidence links PEPCK to C_4_ photosynthesis, none of the positively selected amino acid substitutions are present (Fig. 8b).

### Structural evolution of the *carbonic anhydrase* gene family in the grasses

The first biochemical step in C_4_ photosynthesis is catalyzed by a beta-carbonic anhydrase (CA), which hydrates CO_2_ to produce bicarbonate. The grass lineage contains a locus with multiple tandemly arranged *ca* genes. The *ca* genes comprising this locus in *Z. mays* were previously defined and functionally characterized in *Z. mays* [67]. Rapid Amplification of complementary DNA (cDNA) Ends (RACE) was used to define the *ca1* transcription start site in *D. oligosanthes*, *S. viridis*, and *S. bicolor*, as well as *Brachypodium distachyon* and *Oryza sativa*. These experiments confirmed the expression of a *ca1* transcript in *D. oligosanthes* predicted to be plastid localized, which includes a ~3 kb first intron that is also present in *B. distachyon*, *O. sativa*, and *S. bicolor*. This long transcript predicted to encode a plastid-targeted isoform was also present, but not predicted in *S. viridis*. Transcript data from RACE experiments were used to improve the *ca1* gene annotation for *D. oligosanthes*. Furthermore, RNA-seq data revealed that *ca1* is the most highly expressed of the *ca* gene copies in both C_3_ and C_4_ species.

**Fig. 9:**
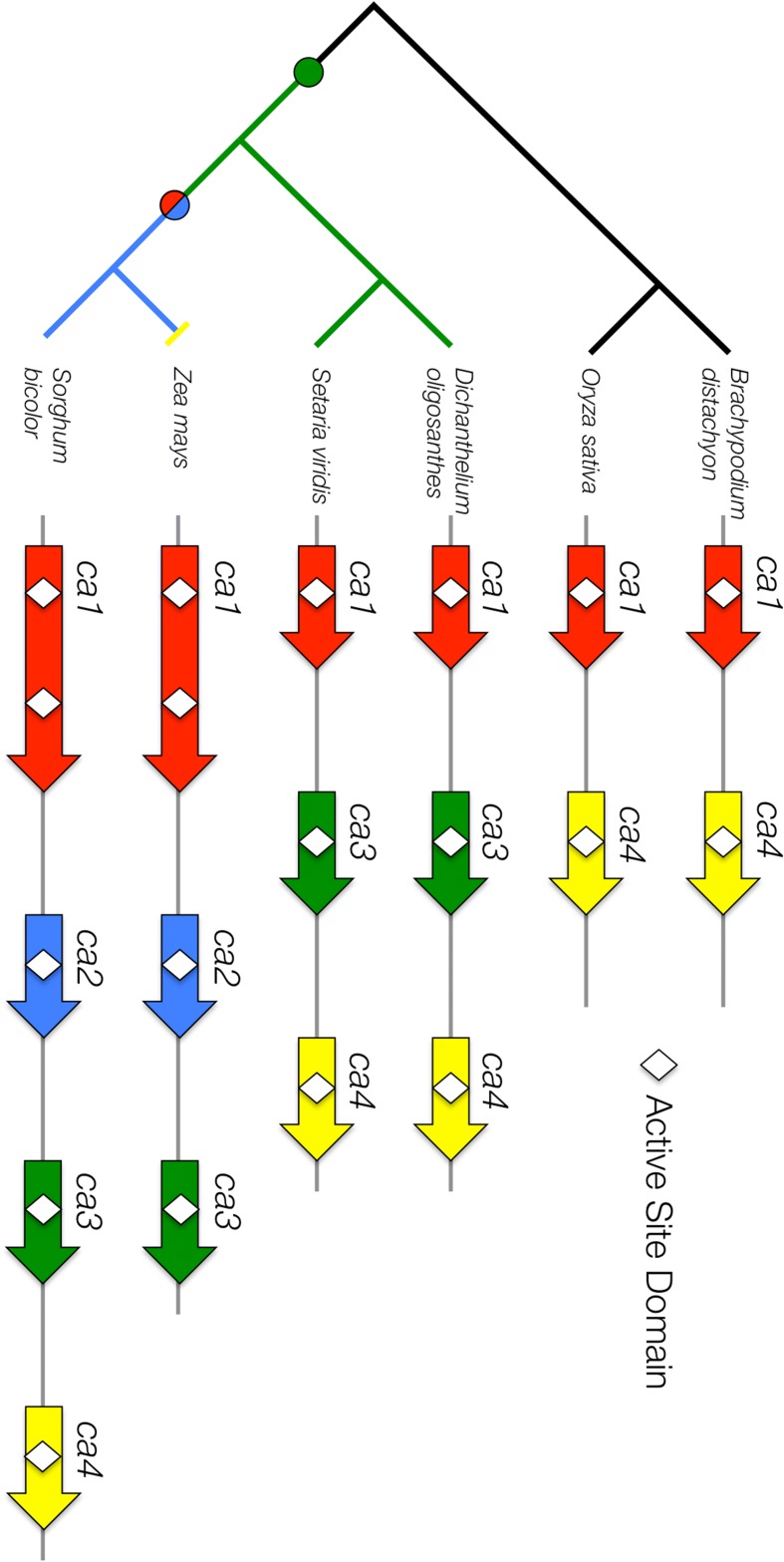
*Carbonic anhydrase* tandem gene duplication. A syntenic region containing tandemly arranged *ca* genes in six grass species. *Lines* show the phylogenetic relationship between the species. *Circles* indicate a duplication event. *Arrows* denote gene copies and *white diamonds* indicate the enzyme active site domain. *Arrow colors* show gene orthology. Not drawn to scale

The number of annotated *ca* genes at this locus varies across grass species, so a comparative genomics approach was used to investigate the evolution of this tandemly arranged *ca* gene cluster (Fig. 9). Using BLAST and synteny comparisons available in CoGe [47, 48], we were able to correct misannotated *ca* genes in several species (see Additional file 5: Carbonic Anhydrase Coding Sequences). Most notably, the *ca4* gene in *O. sativa* was misannotated due to a large transposable element insertion into an intron (LOC_Os01g45290). Additionally, *Osca4* is truncated, lacking the last exon but still retaining the CA active site domain. The *ca4* gene was previously misannotated as a gene fragment in *S. bicolor* [13], but was correctly annotated in the newest release of the genome (version 2.1).

To enable functional comparisons between species, the orthologous/paralogous relationship of the tandemly arranged *ca* genes was examined for grass species with available genomes. *ca1* coding sequences were also obtained for *Panicum virgatum* (switchgrass), *Hordeum vulgare* (barley), *Triticum aestivum* (wheat), and *Musa acuminata* (banana). A maximum-likelihood tree of the *ca* gene copies produced distinct clades and within clades the phylogenetic relationships were preserved (Additional file 1: Figure S4). This analysis provides comprehensive naming of the gene copies across species (Fig. 9).

Based on these comparative analyses, the following model describes the evolution of the *ca* gene family (Fig. 9). The base copy number for the *ca* tandem array in the grasses is two (*ca1* and *ca4*) and in the BEP clade these two tandem gene copies have been retained without modification. In the lineage leading to the panicoid grasses, a duplication of *ca1* generated *ca3* prior to the divergence of the Andropogoneae and Paniceae lineages. The *ca2* gene copy appears to have arisen from a second duplication of *ca1* in the lineage leading to the Andropogoneae tribe and may have been linked with the duplication/fusion event that resulted in two active site domains of *ca1* for species in the same lineage. A deletion of the *ca4* gene is only observed in *Z. mays*. It is currently not clear where *ca4* is expressed or if it is functional; however, the conservation of this gene for 50 million years in both the BEP and (some) PACMAD lineages suggests it likely retained a function for at least some portion of this time. As the number of available grass genomes increases, a better estimate of the timing of gene duplication and loss events will be obtained. As gene duplication provides a foundation for subfunctionalization and neofunctionalization of gene function, comparative genomic approaches that exploit synteny relationships should increase our power to detect signatures of selection.

## Conclusions

The parallel evolution of C_4_ photosynthesis, a complex trait that requires the co-option and redeployment of enzymes from a wide range of biochemical pathways, as well as significant modifications of leaf anatomy, has long been a scientific puzzle. Here, we present the genome sequence of a C_3_ species from within a clade rich in C_4_ origins, the PACMAD grasses. We definitively show that despite having many C_4_ relatives, *D. oligosanthes* uses the C_3_ pathway for carbon fixation by coupling anatomical and physiological characteristics with sequence-based approaches. We demonstrate the usefulness of *D. oligosanthes* as a C_3_ species for investigating C_4_ evolution in the panicoid grasses through a variety of analyses. *D. oligosanthes* is also well placed for future studies of abiotic stress tolerance and adaption to temperate climates. The majority of panicoid grasses are native to tropical or subtropical environments. As a close relative indigenous to the continental United States, *D. oligosanthes* may be able to provide insight into the mechanisms employed by natural selection to develop cold and freezing tolerance in panicoid grass species, knowledge that would be of great value to cold sensitive panicoid crops such as *Z. mays*, *S. bicolor*, and *S. officinarum*. To the best of our knowledge, *D. oligosanthes* is only the third non-cultivated grass species with a sequenced genome, and the first C_3_ grass in the otherwise C_4_-rich PACMAD clade.

Importantly, the results presented here highlight how comparisons among multiple closely related species produce smaller candidate gene lists that may be linked to a trait than pairwise comparisons between individual species. This is especially true when traits have evolved in parallel. Until recently, the time-consuming and expensive process of genome sequencing meant genome assemblies were only available for economically significant plant species (and a small number of widely used models). The uneven evolutionary distribution and small number of model/crop species limited the usefulness of comparative approaches. Today, researchers can select species based on informative phenotypes or phylogenetic positions and generate genomic data as needed. As scientists move beyond pairwise comparisons and begin to seek answers from large clusters of related species with sequenced genomes, new comparative genomics tools and statistical approaches will need to be developed.

## Methods

### Plant material

A wild-grown plant was collected from the Shaw Nature Reserve, in Gray’s Summit, MO. A voucher specimen, *Kellogg 1175*, is deposited at the herbarium of the Missouri Botanical Garden (MO). Locality data are available at http://www.tropicos.org/Specimen/100315254.

### Phylogenetic analysis

Chloroplast gene sequences from *rbcL*, *ndhF*, and *matK* used for the phylogenetic analysis were aligned manually, and the best-fit model was estimated as GTR + G + I by RAxML using all sites [68]. The Bayesian analysis was performed using MrBayes v3.2, with 5 million generations, sampling every 1000 generations. Substitution rates and state frequencies used a Dirichlet prior. The gamma distribution was approximated using four categories.

### Histological analysis

Two-millimeter strips were cut crosswise to the leaf of mature *D. oligosanthes* plants and were fixed for 2 h in 2 % gluteraldehyde in 100 mM, pH 6.8, PIPES buffer at room temperature. The leaves were then washed three times in 100 mM, pH 6.8, PIPES buffer. The leaves were post-fixed in buffered osmium tetroxide for 1.5 h and rinsed three times in water. The leaves were dehydrated in an ethanol/acetone series as follow: 5, 10, 20, 30, 50, 75, 95 % ETOH for 20 min each, followed by 30 min in 100 % ETOH, 15 min in 100 % acetone, and a second 45-min incubation in fresh 100 % acetone. The leaves were infiltrated with Spur’s resin (Cat. no. RT14300, Electron Microscopy Science) dissolved in 100 % acetone as follows: 5 % 12 h, 10 % 12 h, 25 % 24 h, 50 % 24 h, 75 % 24 h, 100 % 24 h. The leaves were then embedded in 100 % resin and incubated at 60 °C for two days. Resin blocks were cut into 1 μM sections using a Leica Ultracut UCT microtome. Slides were stained in 1 % toluidine blue solution (Cat. No. T-140, Spectrum), and then sealed with a No. 1.5 cover glass and Permount (Cat. No. SP15-100, Fisher Scientific). Images were acquired using a Nikon E800 microscope with a 60X PCAN APO, 1.4NA oil immersion phase objective, Digital capture Q-imaging Ratiga 1300 camera, coupled with an LCD color filter, and Q-imaging software.

### Gas exchange measurements

Net rates of CO_2_ assimilation were measured on young, fully expanded leaves using the LI-COR 6400XT gas exchange system (LI-COR Biosciences). Measurements were made at 25 °C and an irradiance of 1500 μmol quanta m^–2^ s^–1^_._ CO_2_ response curves were measured at 25, 19.4, 16.6, 13.5, 7.6, 4.7, 36.7, 60.4, and 140.3 Pa. After gas exchange measurements were complete, leaf material was flash frozen and stored at –80 ° C for enzyme assays.

### Enzyme assays

Approximately 2 cm^2^ leaf material was ground on ice using a mortar and pestle in 1 mL of 50 mM HEPES (pH 7.8), 1 % (v/v) polyvinylpolypyrrolidone, 1 mM EDTA, 10 mM dithiothreitol, 0.1 % (v/v) Triton X-100, and 2 % (v/v) protease inhibitor cocktail (Sigma-Aldrich). Crude extracts were centrifuged at 4 ° C for 1 min at 17,000 g, and supernatant was collected for the CA, Rubisco, and PEPC assays.

CA assays were performed at 25 °C in 2 mL of assay buffer (CO_2_-free 100 mM EPPS-NaOH, pH 8.0, 10 mM DTT). CA activity was measured using a membrane inlet mass spectrometer to measure the rates of ^18^O_2_ exchange from labeled ^13^C^18^O_2_ to H_2_^16^O with a total carbon concentration of 1 mM [69–71]. The hydration rates were calculated from the enhancement in the rate of ^18^O loss over the uncatalyzed rate by applying the non-enzymatic first-order rate constant [72].

Rubisco activity was spectrophotometrically measured in 1 mL of assay buffer (100 mM EPPS-NaOH (pH 8.0), 20 mM MgCl_2_, 1 mM EDTA, 1 mM ATP, 5 mM creatine phosphate, 20 mM NaHCO_3_, 0.5 mM ribulose 1,5-bisphosphate, 0.2 mM NADH) containing coupling enzymes (12.5 units mL^–1^ creatine phosphokinase, 250 units mL^–1^ CA, 22.5 units mL^–1^ phosphoglycerokinase, 20 units mL^–1^ glyceraldehyde-3-phosphodehydrogenase, 56 units mL^–1^ triose isomerase, and 20 units mL^–1^ glycerol-3-phosphodehydrogenase). Rubisco activity was calculated from the consumption of NADH, which was monitored via the change in absorption at 340 nm [73].

PEPC activity was assayed in 1 mL of assay buffer (100 mM EPPS-NaOH (pH 8.0), 20 mM MgCl_2_, 1 mM EDTA, 5 mM NaHCO_3_, 0.2 mM NADH, 5 mM D-glucose-6-phosphate, 12 units mL^–1^ malate dehydrogenase, and 4 mM phosphoenolpyruvate). NADH consumption was monitored at 340 nm [74].

### Modeling

The CO_2_ response curves for C_3_ and C_4_ photosynthesis were modeled according to von Caemmerer (2000). The C_3_ model was matched to gas exchange data from *D. oligosanthes* using a *V*_*cmax*_ = 77 μmol m^–2^ s^–1^ and a *J*_*max*_ = 144 μmol m^–2^ s^–1^. C_4_ photosynthesis was modeled using *V*_*cmax*_ = 35 μmol m^–2^ s^–1^ to have comparable maximum photosynthetic rates to those measured in *D. oligosanthes*. All other modeling parameters were taken from von Caemmerer ([34]; see Tables 2.3 and 4.1).

### Stable isotopes

Dried leaf material was placed in tin capsules and combusted in a hydrogen/carbon/nitrogen elemental analyzer (ECS 4010; Costech Analytical) to determine carbon isotopic composition.

### Growth conditions for physiological measurements

Plants were grown in a controlled-environment growth chamber (Biochambers; GC-16, Winnipeg, Manitoba, Canada) with a 14-h photoperiod and a photosynthetic photon flux density of 500 μmol m^–2^ s^–1^ at leaf height. Relative humidity was maintained at approximately 40 %. Day/night air temperatures were 28 and 18 °C, respectively. Plants were watered as needed and fertilized weekly.

### Genome sequencing and assembly

All DNA used for sequencing was taken from a single F_2_ plant descended from the original collection, *Kellogg 1175*. Following the recommended Allpaths-LG sequencing protocol [43], a 180 bp insert DNA-seq library and 5 kb mate pair DNA libraries were prepared and sequenced at the Cornell University Sequencing Core. Each library was sequenced twice in 2 × 100 bp paired end sequencing lanes. Both libraries were quality trimmed using Trimmomatic [75]. The mate pair libraries were subject to a second quality control step in which polymerase chain reaction duplicate reads (defined as cases where the first 20 base pairs for both forward and reverse reads were identical between independently sequenced clusters) were removed using a simple python script. The resulting dataset was assembled using Allpaths-LG [43], with the ploidy file set to 2 (diploid), and otherwise default settings. Final scaffolding was conducted with SSPACE [44] utilizing long mate pair libraries sequenced on an Illumina MiSeq using 2 × 150 sequencing.

A subset of the total sequencing data (2.3 Gigabytes) was used to assemble the chloroplast genome. SPAdes v.3.1.0 was used to make the initial assembly using the “only-assembler” option with k-mer sizes of 55 and 87. The SPAdes contigs were blasted (blastn) against the *Z. mays* chloroplast genome (NC_001666.2) and mitochondrial genome (NC_007982.1) with an e-value cutoff of 1e-40. The contigs were identified as plastid or mitochondrial using a custom script to determine the optimal e-value and longest single hit length. Plastid-like sequences were then put into Sequencher (Genecodes— 5.2.4). Ends of contigs were identified and were trimmed by 100 base pairs to remove potentially misassembled regions. Twenty base pair sequences from the end of trimmed contigs were used to search all raw reads for exact matching sequence using “grep.” This was repeated with the reverse complement of the sequence. The matching reads were aligned to the contigs in Sequencher and used to extend the contig ends. This process continued until all gaps were closed. The sequence was orientated for the typical representation of large single copy, inverted repeat B, small single copy, and inverted repeat A. Jellyfish v.2.1.3 [51] was used to estimate 20-mer abundance from the reads and these abundance values were matched to the assembled plastome to determine a sliding window coverage across the assembly. No anomalies in coverage were identified and the assembly was considered complete. Annotation of the plastome was completed using DOGMA [52]. A graphical representation of the annotated plastome was created using Circos v.0.66 [53] as implemented in Verdant (verdant.iplantcollaborative.org).

### Gene annotation

Gene annotation was conducted using Maker2 [46]. For each of *O. sativa*, *Brachypodium distachyon*, *S. bicolor*, *Z. mays*, and *S. italica*, the “primaryTranscriptOnly” files for cDNA and protein sequences were downloaded from phytozome and alignments of these files against the *D. oligosanthes* genome assembly were used for the evidence based portion of Maker2’s analysis (Maker2 also incorporates ab initio gene prediction approaches into its final analysis).

### Leaf gradient construction

Plants were sown in MetroMix360 and grown in Conviron BDW-40 controlled chambers under 500 μmol/m^2^/s of light with 12-h light/dark cycles at 31 °C and 22 °C, respectively, and a constant 50 % relative humidity. Leaf tissue was sampled after the second leaf ligule formed but before the third leaf had fully expanded. This corresponds to three weeks after planting for *D. oligosanthes*, and nine days after planting for *S. viridis*. Samples were collected in the morning 2 h after the lights turned on and were immediately flash frozen in liquid nitrogen. Four plants were pooled for each segment replicate of *D. oligosanthes* and ten plants were pooled for each segment replicate of *S. viridis*. TriPure Isolation Reagent (Sigma) was used to extract total RNA following the manufacturer’s recommendations. Libraries were prepared according to Wang et al. [55]. *D. oligosanthes* libraries were sequenced on a HiSeq2000 using a 100 bp paired end run and *S. viridis* libraries were sequenced on a HiSeq2500 using a 100 bp single end run.

### Gene expression analysis

Raw Illumina reads from RNA-seq libraries were subjected to quality trimming using CutAdapt [76] with a minimum quality score of 20 and a minimum read length of 25 bp after trimming. Trimmed reads were aligned to reference genomes (*S. bicolor* v1.4, *D. oligosanthes* v1.0, and *S. italica* v2.1) using [77], allowing splicing over canonical RNA-splice sites, a maximum number of reported alignments per read of 10, a maximum mismatch rate of 3 (allowing up to three SNPs or one SNP and one InDel). FPKM values were calculated using Cufflinks [78].

### Leaf gradient and TF analysis

A minimum FPKM threshold was applied to the Cufflinks output. This threshold stipulates that a gene must have an FPKM value equal to or greater than one in at least one segment of any replicate. Correlation matrices and heat map visualizations were constructed using R software packages (code available upon request). CoGe Blast and CoGe SynFind tools were used to find core C_4_ gene and TF orthologs for the different grass species [47, 48]. Segments 1, 4, 9, and 14 from *Z. mays* and 1, 3, 6, and 9 from *O. sativa* from previously published datasets [55] were compared to the four segment gradient data for *D. oligosanthes* and *S. viridis* generated in this study. Transcription factor leaf gradient expression profiles from the different species were overlaid and compared by eye.

### PEPC amino acid tree

The coding sequence for Do021545.1 contained an alternative splice site and had to be manually curated with raw sequencing reads. Amino acid sequences are listed in Supplementary Material. Amino acid sequences were aligned using the Muscle aligner. The evolutionary history was inferred by using the Maximum Likelihood method based on the JTT matrix-based model. The bootstrap consensus tree inferred from 500 replicates was taken to represent the evolutionary history of the taxa analyzed. Branches corresponding to partitions reproduced in less than 50 % bootstrap replicates are collapsed. The percentage of replicate trees in which the associated taxa clustered together in the bootstrap test (500 replicates) are shown next to the branches. The analysis involved 12 amino acid sequences. All positions containing gaps and missing data were eliminated. There was a total of 863 positions in the final dataset. Evolutionary analyses were conducted in MEGA (version 6.06) [79].

### *ca* gene family

RACE was performed using the GeneRacer kit (Invitrogen, cat#L1502-01) using RNA extracted from leaf tissue. Ten independent clones were sequenced per RACE reaction. *ca* gene coding sequences were aligned using the Muscle codon aligner in MEGA (version 6.06) [79]. *ca1* sequences from *Z. mays* and *S. bicolor* have duplicated active site domains. These gene fusions were treated as two separate genes in the analysis. Alignment gaps were removed and then the alignment was loaded into Cipres Science Gateway (http://www.phylo.org/), where RaxML [68] was used to generate a maximum-likelihood tree.

## Additional files

Additional file 1: Figure S1. Chloroplast genome annotation. **Figure S2** Leaf gradient expression correlation matrices. **Figure S3** PEPC amino acid sequences tree. **Figure S4** *ca* gene tree. (PDF 1990 kb)
Additional file 2: Table S1. Core C_4_ gene orthology. (DOCX 31 kb)
Additional file 3: Table S2. Transcription factor gene list. (DOCX 31 kb)
Additional file 4: PEPC amino acid sequences. (DOCX 35 kb)
Additional file 5: Carbonic anhydrase coding sequences. (DOCX 37 kb)

## Abbreviations

BS: Bundle sheath
CA: Carbonic anhydrase
CCM: Carbon concentrating mechanism
DOGMA: Dual Organellar GenoMe Annotator
M: Mesophyll
NAD-ME: Nicotinamide adenine dinucleotide malic enzyme
NADP-ME: Nicotinamide adenine dinucleotide phosphate malic enzyme
PEPC: Phosphoenopyruvate carboxylase
PEPCK: Phosphoenolpyruvate carboxykinase
RACE: Rapid Amplification of cDNA Ends
rbcL: Rubisco large subunit
Rubisco: Ribulose-1,5-bisphosphate carboxylase/oxygenase
TF: Transcription factor
