# Supplementary material for: The draft genome of the C3 panicoid grass species Dichanthelium oligosanthes
Source: Genome Biol. 2016 Oct 28;17:223. doi: 10.1186/s13059-016-1080-3 (PMC5084476; doi:10.1186/s13059-016-1080-3)

Figure S1: Chloroplast genome annotation.

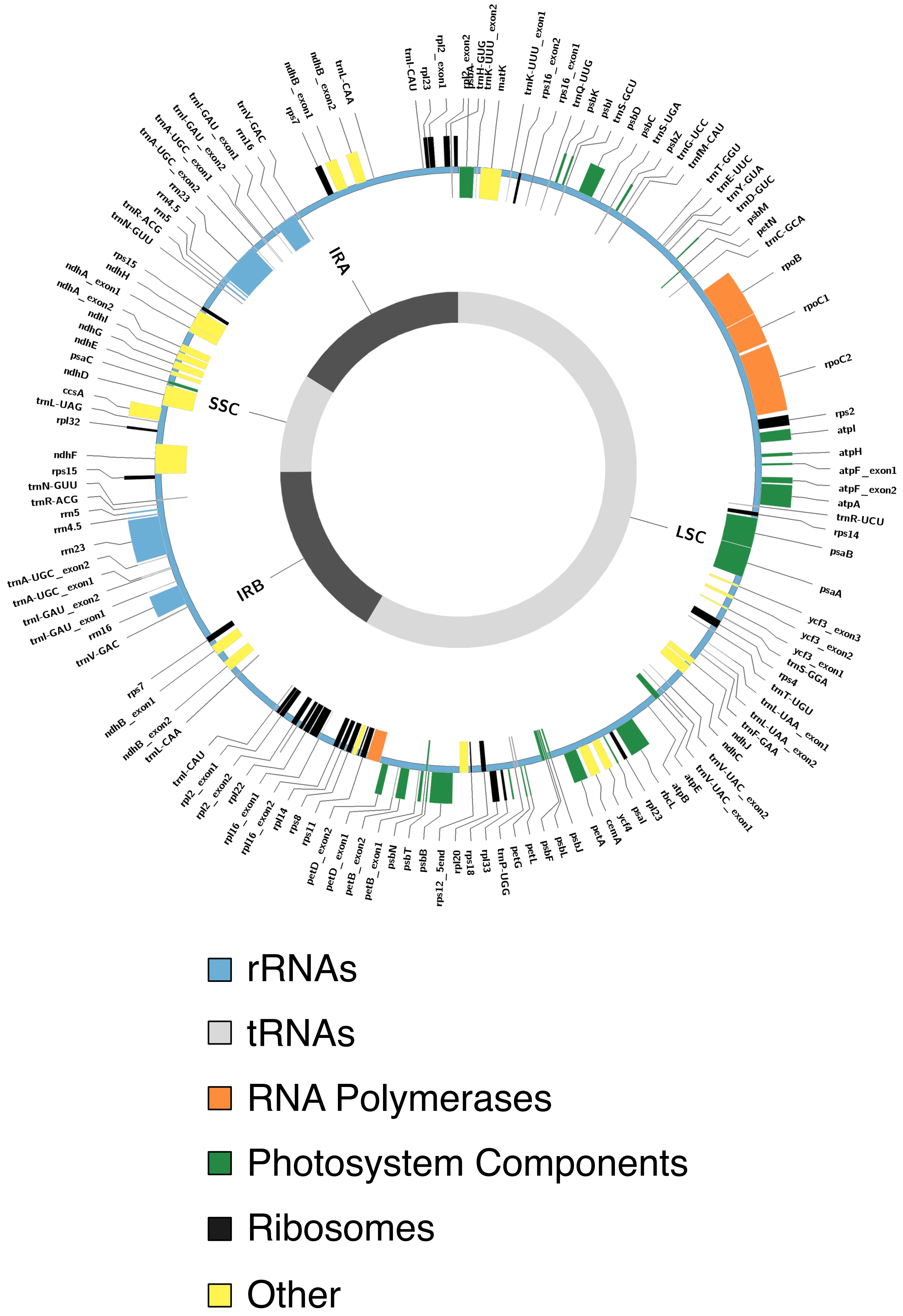

Figure S2: Leaf gradient expression correlation matrices.

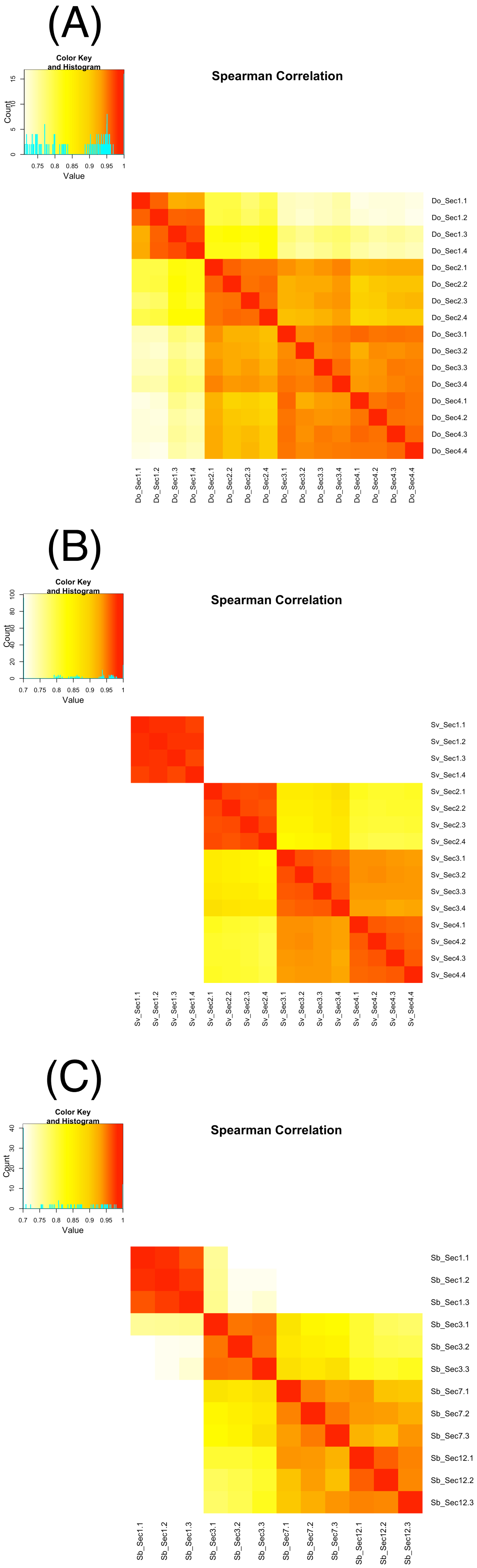

Figure S3: PEPC amino acid sequences tree.

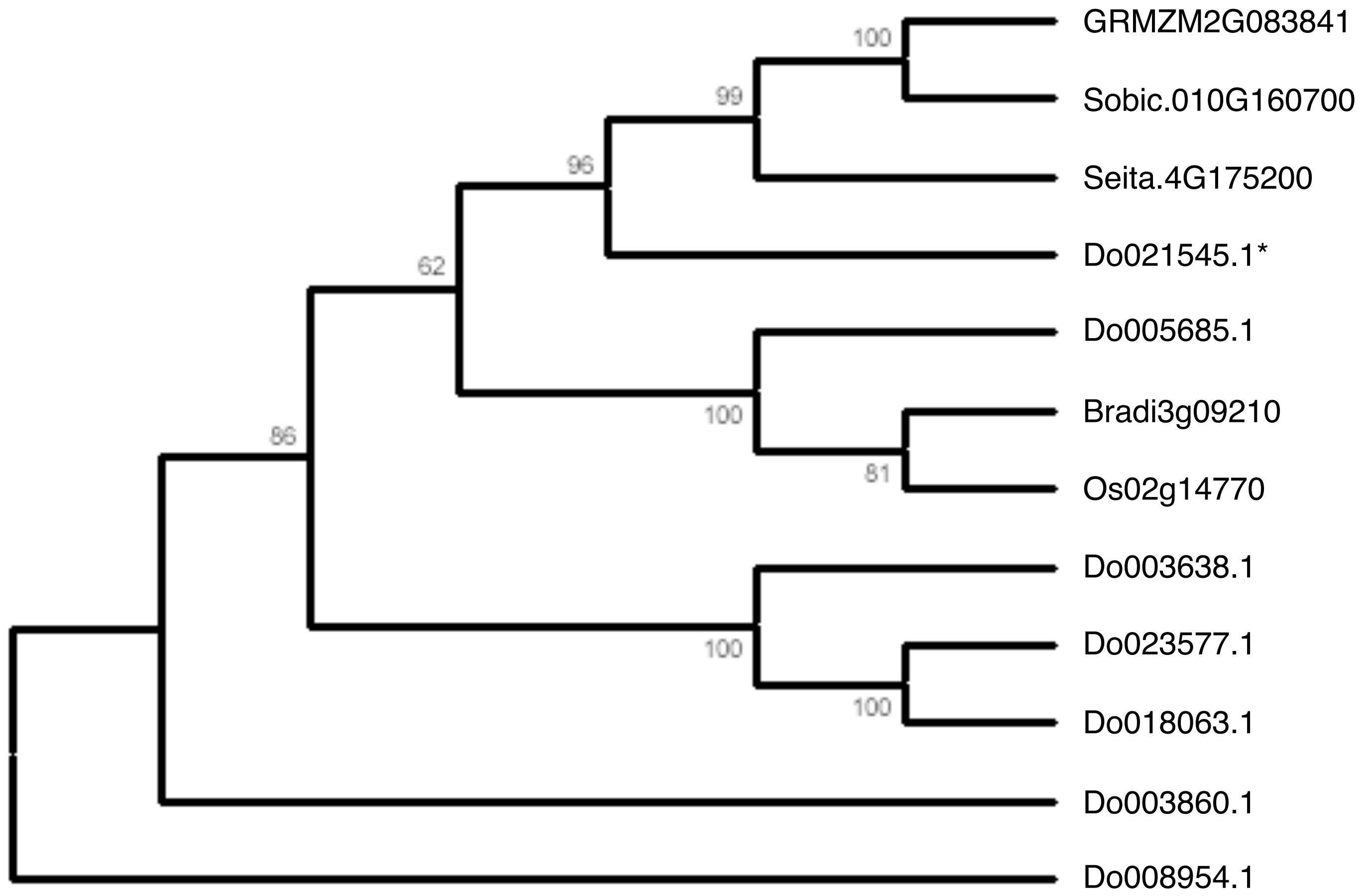

Figure S4: *Carbonic anhydrase* gene tree.

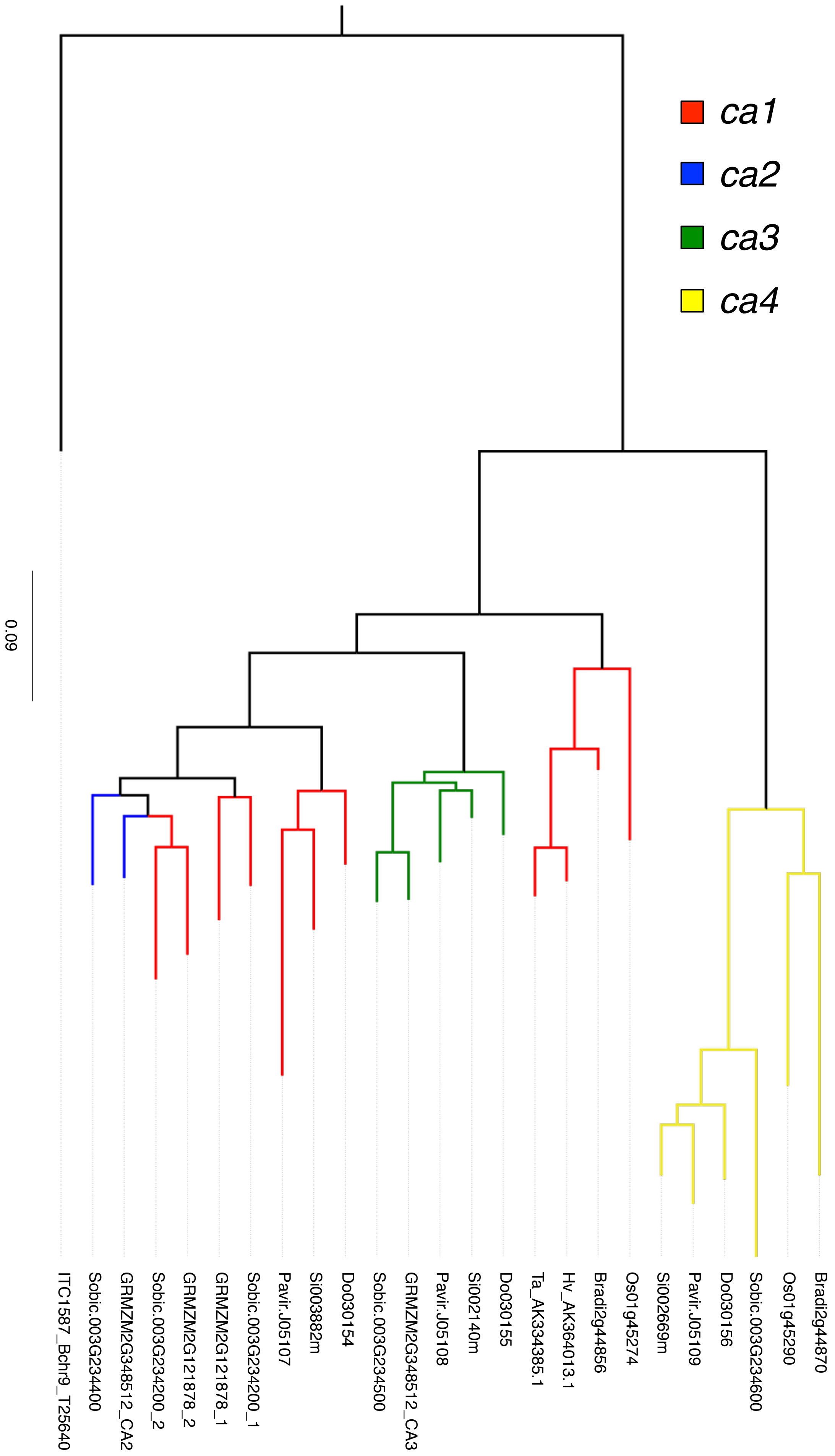

Supplement: Additional file 1: Figure S1. — Chloroplast genome annotation. Figure S2 Leaf gradient expression correlation matrices. Figure S3 PEPC amino acid sequences tree. Figure S4 ca gene tree. (PDF 1990 kb) [file 13059_2016_1080_MOESM1_ESM.pdf]
