# Supplementary material for: The draft genome of the C3 panicoid grass species Dichanthelium oligosanthes
Source: Genome Biol. 2016 Oct 28;17:223. doi: 10.1186/s13059-016-1080-3 (PMC5084476; doi:10.1186/s13059-016-1080-3)
Supplement: Additional file 2: Table S1. — Core C4 gene orthology. (DOCX 31 kb) [file 13059_2016_1080_MOESM2_ESM.docx]

**Table S1 Core C_4_ gene orthology.**

| **Gene** | **Abbreviation** | ***D. oligosanthes*** | ***S. italica*** | ***S. bicolor*** |
| --- | --- | --- | --- | --- |
| Carbonic Anhydrase 1 | CA | Do030154.1 | Si003882m.g | Sb03g029170 |
| Phosphoenopyruvate Carboxylase | PEPC | Do021545.1 | Si005789m.g | Sb10g021330 |
| NADP-malate dehydrogenase | NADP-MDH | Do003942.1 | Si013632m.g | Sb07g023920 |
| Pyruvate Orthophosphodikinase | PPDK | Do015534.1 | Si021174m.g | Sb09g019930 |
| NADP-malic enzyme | NADP-ME | Do024386.1 | Si000645m.g | Sb03g003230 |
| Phosphoenolpyruvate Carboxykinase | PEPCK | Do007037.1 | Si034404m.g | Sb01g040720 |
