# Supplementary material for: The draft genome of the C3 panicoid grass species Dichanthelium oligosanthes
Source: Genome Biol. 2016 Oct 28;17:223. doi: 10.1186/s13059-016-1080-3 (PMC5084476; doi:10.1186/s13059-016-1080-3)
Supplement: Additional file 3: Table S2. — Transcription factor gene list. (DOCX 31 kb) [file 13059_2016_1080_MOESM3_ESM.docx]

**Table S2 Transcription factor gene list.**

| ***Z. mays*** | ***S. bicolor*** | ***S. italica*** | ***D. oligosanthes*** | ***O. sativa*** |
| --- | --- | --- | --- | --- |
| GRMZM2G147152 | Sb04g023990 | Si017567m.g | Do028914.1 | LOC_Os11g10130 |
| GRMZM2G040481 | Sb04g023730 | Si017107m.g | Do029416.1 | N/A |
| GRMZM2G098986 | N/A | N/A | Do013591.1 | N/A |
| GRMZM2G130149 | Sb08g018580 | Si023104m.g | Do007228.1 | LOC_Os06g49740 |
| GRMZM2G119999 | Sb07g029150 | Si040014m.g | Do026738.1 | LOC_Os11g01140 |
| GRMZM2G140355 | Sb10g023420 | Si018524m.g | Do020983.1 | LOC_Os06g39960 |
| GRMZM2G061906 | Sb09g022280 | Si023133m.g | Do013698.1 | LOC_Os01g59160 |
| GRMZM2G054252 | Sb07g025990 | Si014408m.g | Do007526.1 | LOC_Os01g21180 |
