# Supplementary material for: The draft genome of the C3 panicoid grass species Dichanthelium oligosanthes
Source: Genome Biol. 2016 Oct 28;17:223. doi: 10.1186/s13059-016-1080-3 (PMC5084476; doi:10.1186/s13059-016-1080-3)
Supplement: Additional file 4: — PEPC amino acid sequences. (DOCX 35 kb) [file 13059_2016_1080_MOESM4_ESM.docx]

**PEPC amino acid sequences.**

>Bradi3g09210

MAAPAGKAAIERHQSIDAQLRLLAPGKVSEDDKLVEYDALLVDRFLDILQDLHGPNLREFVQDCYELSAEYENDRDEARIAELGSKLTSLSPADSIVVSSSFSHMLNLANLAEEVQIAFRRRIKLKRGDFGDEGSAPTESDIEETLKRLVSDLGKSREEVFDALKSQTVDLVFTAHPTQSVRRSLLQKHGRIRNCLRQLYAKDITADDKQELDEALQREIQAAFRTDEIRRTPPTPQDEMRAGMSYFHETIWKGVPKFLRRIDTALKNIGINERLPYNAPLIQFSSWMGGDRDGNPRVTPEVTRDVCLLARMMAANLYFSQIEDLMFELSMWRCSDELRVRADELHRSSKKSAKHYIEFWKQVPPNEPYRVILGDVRDKLYYTRERSRHILTTGISDIPEESTFTNVEQFLEPLELCYRSLCACGDKPIADGSLLDFLRQVSTFGLALVKLDIRQESDRHTDVLDAITTHLGIGSYAEWSEEKRQDWLLSELRGKRPLFGSDLPQTEEVADVLGTFHVLAELPADCFGAYIISMATAPSDVLAVELLQRECHIQQPLRVVPLFEKLADLEAAPAAVARLFSIDWYMNRINGKQEVMIGYSDSGKDAGRLSAAWQMYKAQEELIKVAKHYGVKLTMFHGRGGTVGRGGGPSHLAILSQPPDTIHGSLRVTVQGEVIEHSFGEEHLCFRTLQRFTAATLEHGMHPPISPKPEWRALMDEMAVVATKEYRSIVFQEPRFVEYFRSATPETEYGRMNIGSRPSKRKPSGGIESLRAIPWIFAWTQTRFHLPVWLGFGAAFKHIMQKDIRNIHTLREMYNEWPFFRVTLDLLEMVFAKGDPGIAALYDKLLVAEDLQSFGEQLRNNFEETKQLLLQVAGHKDVLEGDPYLKQRLRLRESYITTLNVCQAYTLKRIRDPSFEVTPQQPPLSKEFTDEKEPAELVQLNRGSEYAPGLEDTLILTMKGIAAGMQNTG

>GRMZM2G083841

MASTKAPGPGEKHHSIDAQLRQLVPGKVSEDDKLIEYDALLVDRFLNILQDLHGPSLREFVQECYEVSADYEGKGDTTKLGELGAKLTGLAPADAILVASSILHMLNLANLAEEVQIAHRRRNSKLKKGGFADEGSATTESDIEETLKRLVSEVGKSPEEVFEALKNQTVDLVFTAHPTQSARRSLLQKNARIRNCLTQLNAKDITDDDKQELDEALQREIQAAFRTDEIRRAQPTPQDEMRYGMSYIHETVWKGVPKFLRRVDTALKNIGINERLPYNVSLIRFSSWMGGDRDGNPRVTPEVTRDVCLLARMMAANLYIDQIEELMFELSMWRCNDELRVRAEELHSSSGSKVTKYYIEFWKQIPPNEPYRVILGHVRDKLYNTRERARHLLASGVSEISAESSFTSIEEFLEPLELCYKSLCDCGDKAIADGSLLDLLRQVFTFGLSLVKLDIRQESERHTDVIDAITTHLGIGSYREWPEDKRQEWLLSELRGKRPLLPPDLPQTDEIADVIGAFHVLAELPPDSFGPYIISMATAPSDVLAVELLQRECGVRQPLPVVPLFERLADLQSAPASVERLFSVDWYMDRIKGKQQVMVGYSDSGKDAGRLSAAWQLYRAQEEMAQVAKRYGVKLTLFHGRGGTVGRGGGPTHLAILSQPPDTINGSIRVTVQGEVIEFCFGEEHLCFQTLQRFTAATLEHGMHPPVSPKPEWRKLMDEMAVVATEEYRSVVVKEARFVEYFRSATPETEYGRMNIGSRPAKRRPGGGITTLRAIPWIFSWTQTRFHLPVWLGVGAAFKFAIDKDVRNFQVLKEMYNEWPFFRVTLDLLEMVFAKGDPGIAGLYDELLVAEELKPFGKQLRDKYVETQQLLLQIAGHKDILEGDPFLKQGLVLRNPYITTLNVFQAYTLKRIRDPNFKVTPQPPLSKEFADENKPAGLVKLNPASEYPPGLEDTLILTMKGIAAGMQNTG

>LOC_Os02g14770

MAAAAGKAAMERHQSIDAQLRLLAPGKVSEDDKLVEYDALLVDRFLDILQDLHGPHLREFVQECYELSAEYENDRDEARLDELGRKLTSLPPGDSIVVSSSFSHMLNLANLAEEVQIAHRRRIKLKRGDFADEASAPTESDIEETLKRLVTQLGKSREEVFDALKNQTVDLVFTAHPTQSVRRSLLQKHGRIRNCLRQLYAKDITADDKQELDEALQREIQAAFRTDEIRRTPPTPQDEMRAGMSYFHETIWKGVPKFLRRIDTALKNIGINERLPYNAPLIQFSSWMGGDRDGNPRVTPEVTRDVCLLARMMAANLYFSQIEDLMFELSMWRCSDELRIRADDLHCSSRKAAKHYIEFWKQIPPNEPYRVILGGVRDKLYYTRERTRHLLTTGVSEIPEEATFTNVEEFLEPLELCYRSLCACGDKPIADGSLLDFLRQVSTFGLALVKLDIRQESDRHTDVLDAITTYLGIGSYAEWSEEKRQDWLLSELRGKRPLFGPDLPQTEEIADVLGTFHVLAELPADCFGAYIISMATAPSDVLAVELLQRECHIKQPLRVVPLFEKLADLEAAPAAVARLFSIDWYMNRINGKQEVMIGYSDSGKDAGRLSAAWQMYKAQEELVKVAKHYGVKLTMFHGRGGTVGRGGGPSHLAILSQPPDTIHGSLRVTVQGEVIEHSFGEEHLCFRTLQRFTAATLEHGMHPPISPKPEWRALMDEMAVVATKEYRSIVFKEPRFVEYFRSATPETEYGRMNIGSRPSKRKPSGGIESLRAIPWIFAWTQTRFHLPVWLGFGGAFKHIMQKDIRNIHTLKEMYNEWPFFRVTLDLLEMVFAKGDPGIAALYDKLLVAGDLQSFGEQLRNNFEETKQLLLQVAGHKDILEGDPYLKQRLRLRESYITTLNVCQAYTLKRIRDPSFEVMSQPALSKEFVDSNQPAELVQLNAASEYAPGLEDTLILTMKGIAAGMQNTG

>Seita.4G175200

MASKPVEKHHSIDAQLRLLAPGKVSEDDKLVEYDALLIDRFLDIFQDLHGPNIREFVQECYEVAAEYERDRDAAKLSELGSRLTKLSPNDAIVVASSFSNMLNLTNLAEEVQLAHLRRNKLKRGDFADEGFAATESDIEETLKRLVTELGKSKEEVFDALKNQTVDLVFTAHPTQSVRRSLLQKHARIRNCLTQLYAKDITEDDKQELDEALQREIQACFRTDEIRRAQPTPQDEMRYGMSYFHETIWKGVPKFLRRVDTALKNIGIDERLPYNAPLIQFSSWMGGDRDGNPRVTPEVTRDVCLLARMMASNLYFSQIEELMFELSMWRCNDELRARAEELHASSQKISKHYIEFWRQLPANEPYRVVLGYVRDKLYSTRERSRHLLTSGFSDIPEDSAFKNVEEFLEPLELCYKSLCDCGDKTIADGSLLDFMRQVSTFGLSMVKLDIRQESERHTDVIDAITTHLGIGSYREWSEEKRQEWLLSELRGKRPLLSKDMPQTEEIADVLGCFHVLAELPRDSFGPYIISMATAPSDVLAVELLQRECHVKQPLPVVPLFEKLADLQSAPASIERLFSLDWYMNRIGGKQQVMVGYSDSGKDAGRLSAAWHLYKAQEAMAKVAKRYGVKLTMFHGRGGTVGRGGGPTHLAILSQPPDTINGSLRVTVQGEVIETSFGEEHLCFRTLQRFTAATLEHGMHPPVSPKPEWRALMDEIAAVATDEYRSVVMREPRFVEYFRSATPETEYGRLNIGSRPAKRKPKGGIESLRAIPWIFSWTQTRFHLPVWLGFGAAFKHAMKKDIKNFQMLKEMYNEWPFFRVTLDLLEMVLAKGDPTIAGLYDQLLVADELKPFGEQLRNNYVETEKLILQVAGHKEILESDPGLKQQLRLRDPYITILNVWQAYTLKRIRDPNFKVTPQPPLSKEFADENQPRGIVKLNPASEYGPGLEDTLILTMKGIAAGMQNTG

>Sobic.010G160700

MHAFPIPRAHADHTLANSPSLFEATGRRPALINQLAAEEQHEQHAAPRSNHLQLRSRFPAHSPAAAAMASERHHSIDAQLRALAPGKVSEELIQYDALLVDRFLDILQDLHGPSLREFVQECYEVSADYEGKKDTSKLGELGAKLTGLAPADAILVASSILHMLNLANLAEEVELAHRRRNSKLKHGDFSDEGSATTESDIEETLKRLVSLGKTPAEVFEALKNQSVDLVFTAHPTQSARRSLLQKNARIRNCLTQLSAKDVTVEDKKELDEALHREIQAAFRTDEIRRAQPTPQDEMRYGMSYIHETVWNGVPKFLRRVDTALKNIGINERLPYDVPLIKFCSWMGGDRDGNPRVTPEVTRDVCLLSRMMAANLYINQVEDLMFELSMWRCNDELRARAEEVQSTPASKKVTKYYIEFWKQIPPNEPYRVILGAVRDKLYNTRERARHLLATGFSEISEDAVFTKIEEFLEPLELCYKSLCECGDKAIADGSLLDLLRQVFAFGLSLVKLDIRQESERHTDVIDAITTHLGIGSYRSWPEDKRMEWLVSELKGKRPLLPPDLPMTEEIADVIGAMRVLAELPIDSFGPYIISMCTAPSDVLAVELLQRECGIRQTLPVVPLFERLADLQAAPASVEKLFSTDWYINHINGKQQVMVGYSDSGKDAGRLSAAWQLYVAQEEMAKVAKKYGVKLTLFHGRGGTVGRGGGPTHLAILSQPPDTINGSIRVTVQGEVIEFMFGEENLCFQSLQRFTAATLEHGMHPPVSPKPEWRKLMEEMAVVATEEYRSVVVKEPRFVEYFRSATPETEYGKMNIGSRPAKRKPGGGITTLRAIPWIFSWTQTRFHLPVWLGVGAAFKWAIDKDIKNFQKLKEMYNEWPFFRVTLDLLEMVFAKGDPGIAGLYDELLVAEELKPFGKQLRDKYVETQQLLLQIAGHKDILEGDPYLKQGLRLRNPYITTLNVFQAYTLKRIRDPSFKVTPQPPLSKEFADENKPAGLVKLNPASEYPPGLEDTLILTMKGIAAGMQNTG

>Do021545.1*

MASFKPPNPVERHQSIDAQLRLLAPGKVSEDDKLIEYDALLVDRFLDILQDLHGPSLREFVQECYELSAEYEGDRDAARLSELGDRLTGLAPADAIVVASSFSHMLNLANLAEEVQIAHRRRNKLKRGDFADEASATTESDIEETLKRLVTELGKSREEVFDALKNQTVDLVFTAHPTQSIRRSLLQKHARIRNCLTQLYAKDITADDKQELDEALQREIQAAFRTDEIRRTQPTPQDEMRAGMSYFHETIWKGVPKFLRRVDTALKNIGIDERLPYNAPLIQFSSWMGGDRDGNPRVTPEVTRDVCLLARMMAANLYFSQIEELMFELSMWRCNDELRVRAEELHRASRKAAKHYIEFWKQIPPNEPYRVILGYVRDKLYYTRERSRHLLTTGFSEIPEDSAFINVEEFLEPLELCYRSLCACGDKTIADGSLLDFLRQVSTFGLSLVKLDIRQESERHTDVLDAITTHVGVGSYREWPEEKRQEWLLSELRGKRPLLGPDLPQTEEVADVLGTFRVLAELPPDSFGAYIISMATAPSDVLAVELLQRECHVRHPLRVVPLFEKLADLEAAPAAVARLFSVDWYMERINGKQEVMIGYSDSGKDAGRLSAAWQLYKAQEELIQVAKRYGVKLTMFHGRGGTVGRGGGPTHLAILSQPPDTIHGSLRVTVQGEVIEHSFGEEHLCFRTLQRFTAATLEHGMHPPVSPKPEWRALLDELAVVATEEYRSIVFREPRFVEYFRSATPETEYGRMNIGSRPSKRKPSGGIESLRAIPWIFAWTQTRFHLPVWLGFGAAFKHAIKKDIRNIQMLREMYNEWPFFRVTLDLLEMVFAKGDPGIAGLYDELLVTGDLKPFGEQLRNNHLDTQQLLLQVAGHKDILEGDPYLKQRLRLRDPYITTLNVCQAYTLKRIRDPSFQITAQRPLSKEFADENQPAGLVKLNPASEYAPGLEDTLILTMKGIAAGMQNTG

>Do023577.1

MAGKLEKMASIDAQLRMLAPAKLSEDDKLVEYDALLLDRFLDILQDLHGEDLREMVQECYEIAAEYERKRDSEKLDELGNMLTSLDPGDSIVMAKAFSHMLNLANLAEEVQIAYRRRIKLKKGDFADENSALTESDIEETFKRLVVDLKKSPAEVFDALKSQTVDLVLTAHPTQSVRRSLLQKHSRIRNCLVQLYSKDITPDDKQELDEALQREIQGAVRTDEIKRTQPTPQDEMRAGMSYFHETIWKGVPKFLRRVDTALKNIGIDERMPYNAPLIQFSSWMGGDRDGNPRVTPEVTRDVCLLARMMAANLYCSKIEDLMFELSMWRCNDDLRARADELHRSTKKDAKHYIEFWKKVPLSEPYRVILGDVRDKLYNTRERARQLLSSGYSDIPEESTLTNVEQFLEPLELCYRSLCACGDRVIADGSLLDFLRQVSTFGLCLVRLDIRQESDRHTDVLDAITTYLGIGSYREWSEERRQEWLLSELNGKRPLFGPDLPTTDEIADVLDTFRVIAELPADNFGAYIISMATAPSDVLAVELLQRECQVKTPLRVVPLFEKLADLEGAPAALARLFSVDWYRERINGKQEVMIGYSDSGKDAGRLSAAWQLYKAQEELIKVAKQFGVKLTMFHGRGGTVGRGGGPTHLAILSQPPDTIHGSLRVTVQGEVIEQSFGEEHLCFRTLQRFTAATLEHGMHPPISPKPEWRALLDEMAVVATKEYRSIVFQEPRFVEYFRLATPEMEYGRMNIGSRPSKRKPSGGIESLRAIPWIFAWTQTRFHLPVWLGVGAAFKHILEKDIRNLHMLQEMYNEWPFFRVTIDLVEMVFAKGDPGIAALYDKLLVSSELWPLGEKLRANYEETKRLLLQVAGHKDLLEGDLYLKQRLRLRDAYITTLNVCQAYTMKRIRDPDYHVTLRPHLSKEIMDWNKPAAELVKLNPTSEYAPGLEDTLILTMKGIAAGMQNTG

>Do018063.1

MAALGAKVERLSSIDAQLRMLVPGKLSEDDKLIEYDALLLDRFLDILQDLHGDDLKEMVQECYEVAAEYETKHDLQKLDELGKMITSLDPGDSIVIAKSFSHMLNLANLAEEVQIAYRRRIKLKKGDFADENSAMTESDIEETLKRLVFDLKKSPAEVFDALKSQTVDLVLTAHPTQSVRRSLLQKHSRIRNCLVQLYSKDITPDDKQELDEALQREIQAAFRTDEIRRTQPTPQDEMRAGMSYFHETIWKGVPKFLRRVDTALKNIGIDERVPYNAPLIQFSSWMGGDRDGNPRVTPEVTRDVCLLARMMAANLYCSQIEDLMFELSMWRCSDELRIRADELHRSTKKDAKHYIEFWKKVPPNEPYRVILSDVRDKLYNTRERSRELLSSGHSDVPKEATLTSVEQLLEPLELCYRSLCACGDRVIADGSLLDFLRQVSTFGLSLVRLDIRQESDRHTDVLDAITTYLGIGSYRKWPEERRQEWLLSELNGKRPLFGSDLPKTEEVADVLETFHVIAELPADNFGAYIISMATSPSDVLAVELLQRECHVKTPLRVVPLFEKLADLEAAPAALSRLFSIDWYRQRINGKQEVMIGYSDSGKDAGRLSAAWQLYKAQEELIKVAKDFGVKLTMFHGRGGTVGRGGGPTHLAILSQPPDTIHGSLRVTVQGEVIEQSFGEEHLCFRTLQRFTAATLEHGMHPPNAPKPEWRALLDEMAVVATEEYRSIVFKEPRFVEYFRLATPETEYGRMNIGSRPSKRKPSGGIESLRAIPWIFAWTQTRFHLPVWLGFGAAFKHVLQKDIRNLHMLQEMYNEWPFFRVTIDLIEMVFAKGNPGIAALYDKLLVSEDLQPLGEKLRANYEETQKLLLQVAGHRDLLEGDPYLKQRLRLRDAYITTLNVCQAYTLKRIRDPDYHVALRPHLSKEIMDSSKPAAELVKLNPASEYAPGLEDTLILTMKGIAAGLQNTG

>Do008954.1

MGTAAARGRRLLPLGLALLLVVLRGCAGAGQGEEDGASDSPAAETAPMEEKERAALYAAIESFVGKGWNGSGLYPDPCGWSPIQGVSCDLFNGLWYPTAISIGPVLDNSLQCAPDPKFSPQLFDLRRLRTLSFYSCFPASNPTAIPTASWEKLSGSLETLEFRTNPGLTGAIPASLGRLASLQSLVLVENNLTGPVPGELGALLKLRRLVLSGNGLSGPIPATLGNARHDELLIVDLSKNSLTGSLPSSLGGLRGLLKMDLSNNQLKGSIPPELGGLKSLTLLDLRNNSLTGGLPQFVQGMASLQDLLLSNNPLGGTLPASGWEKLPSLATLDLSNLGLAGAIPESMASLSGLRFLALDHNRLTGAVPPGLARLPSIGALYLNGNNLTGALGFAPGFYQRMGRRFASWDNPGLCYNVAAVDAAHAPAGVVVCKDLQGPSAGGGGGTQDGRKPEASSSLMASSSLGFSAARVRGFWSMVMAQGTVAALLVSVWPLGMLVQVHEKAPAAEVGENSERRGRRAGGRMPDSTDDIAEGISFQAFEDDCRLLATLLHDVLLRELGPRFIQILERNRILAQSAVSMRAAGMEDTAAVVERQLEADLAAMTLDDALCLARAFSHYLNLMGIAETHHRVRRARNVEQLSKSCDDIFDKLIQSGVPPEQLYDTVCKQEVEIVLTAHPTEINRRTLQYKHLRVAHLLEFNARPVLSHEDKEMLIEDLVREITAIWQTDELRRHKPTPVDEARAGLHIVEQSLWKAVPHYLRRVSNALKKHTGSPLPLTCTPIKFGSWMGGDRDGNPNVTAKVTRDVSLLSRWMAINLYIRELDNLSFELSVKRCSDKVASLANEILLKESASEDLKANTWSQTAPQNNAKLHHNLPLPAHLPSGADFPSCTECSDGESQIRMINLPRNPSHPGALNLTEKFEDSPLPSPTGRQSQIGRTPSGGQLRKLFKKSHIPRSSSFRKLLEPSISDRPGITPYRVVLGNVKEKLVKTRRRLELLLEDLPCDYDTEEYCETSDQLLEPLLLCYQSLLLIFVLATSEKLMQSFPLLQESSRHTEALDAVTSYLDLGVYSEWDEEKKLDFLTRELKGKRPLVPPNIEVAADVKEVLDTFKVAAELGSDSLGAYVISMASNASDVLAVELLQKDARLAVSGDLGRPCPGGTLRVVPLFETVKDLRDAGAAIRKLLSIDWYREHVIKNHNGHQEVMVGYSDSGKDAGRFTAAWELYKAQEDVVAACNEFGIKVTLFHGRGGSIGRGGGPTYLAIQSQPPGSVMVSVSYPVQSFRMCCFRRKEVWPPQGTLRSTEQGEMVQAKFGLPQTAVRQLEIYTTAVLLATLRPPQPPRDPNWRHVMEEISRVSCAHYRRTVYEDPEFITYFQEATPQAELGYLNIGSRPAKRKPSGGISSLRAIPWVFAWTQTRLVLPAWLGVGTGLQDALDRGHGEELRAMYAEWPFFQSTVDLIEMVTAKADAPMAAHYEEMLVAPERRAVGGELRRELARTERCVLAVSGHVKLTAHNRSLRRLIESRLAYLNPINMLQVECSLACWAPMASSPSPSPGTIRATLPPSTAIPSPVTTTPTPASPAPVTVPNATPADPPSSPAAPPPLTPASTPPPQLASPPPSSPPPPDAVPPPPVVIASPPPAPAAVVPPPSPPVAVPPPPTPAAPPKASPILPPAAASPPPSNLPAPNPPADPTPPTVVQPPPPRHRRPPRSPGTQPEPPPLAPPPSAIPVKPSPTSPSPNSGDPLIPTPTSPSPPGMTPSTPGSGSPSVPSPATAVDPVSPVTTGDRGSNKSSSSATQSSSSVSSGEMSSGAKAGIGVVVAILVLSLVGAVFWYKKKRRRVHGYHAGFIMPSPASTPTQVLGYSAKTNFSAGSPESKDSMPEFSMSNCRFFAYEELYQITNGFSSQNLLGEGGFGSVYKGCLADGKQVAVKKLKEGGGQGEREFHAEVEIISRVHHRHLVSLVGYCISDDQRLLVYDFVPNNTLHYHLHGRGVPVLEWPARVKIAAGSARGIAYLHEDCHPRIIHRDIKSSNILLDNNFEAQVADFGLARLALDACTHVTTRVMGTFGYLAPEYASSGKLTERSDVFSFGVVLLELITDVYKRKNYNEVEMFRMIEAAAACIRHSASRRPKMSQVVRVLDSLADVDLTNGIQPGKSEMFNVANTAEIRLFQRMAFGSQDFTTDFSQSSWNSQSRGLDASGQSRGPDASGSRPL

>Do003638.1

MSLSPSQETRYSIRNVFCRVDLPPPRPRLGLARKGVNLFQESGARGEREGLGEREMARNAVDKATSIDAQLRLLAPQKLSDDDKLVEYDALLLDRFLDILQDLHGEDIRETVQECYELAAEYESKLDPKMLGEIGNVLTNLDPGDSIVITKSFSHMLILANLAEEVQIAYRRRIKLKKGDFVDENSATTESDIEETLKRLVHQLKKSPMEVFDALKNQTVDLVLTAHPTQSVRRSLLQKHGRIRDCLSQLYAKDITPDEKLELDEALQREIQAAFRTDEIRRAPPTPQDEMRAGMSYFHETIWKGVPKFLRRVDTALKNIGINERVPYNAPLIQFSSWMGGDRDEFWKQVPPSEPYRVILSDVRDKLYNTRERARHLLASGFSEIPEEATFTDVEQFLELLELCYRSLCACGDRSVADGSLLDFLRQVSTFGLSLVRLDIRQESDRHTDVMDVITEYLGIGSYHEWPEEKRQEWLLSELNGKRPLFGPDLPKSDEIADVLETFHVLAELPSDSFGAYVISMATAPSDVLAVELLQRECHVKKPLRVVPLFEKLADLEAAPAALARLFSVEWYRNRISGKQEVMIGYSDSGKDAGRFSAAWQLYKAQEELIKVAKLYGVKLTMFHGRGGTVGRGGGPTHLAILSQPPETIHGSLRVTVQGEVIEQSFGEEHLCFRTLQRFTAASLEHGMHLPISPKPEWRALMDEMAIVATKEYRSIVFEEPRFVEYFRLSLTFHSCFCGLRIFLASPEDYLRYKIVSISTVMSLLLVATPEMEYGRMNIGSRPSKRKPSGGIESLRAIPWIFAWTQTRFHLPVWLGFGAAFKHVLDKDIRNLQTLQEMYNQWPFFRVTIDLVEMVFAKGDPGIAALYDKLLVSEDLWSFGERLRANYEETKQLLLQVAGHKDLLEGDPYLKQRLRIRDSYITALNVCQAYTLKRIRDPGFQVNPRPHLSKDIMDAGKPAAELVKLNTTSEYAPGLEDTLILTMKGIAAGMQNTG

>Do003860.1

MVSLRLAPFTLLVELPASRRRLKLARCTVRRARSVPSSAAAKAVGEVAGPASASVAEERRSIDAHLRQLAVSADDRLVDYETLLVARFLDILQDLHGGDFRQVVEECLRLSGEYHGGGDPARLDELGALLTCLDVGDAIMVVSSFSHMLNLANIAEEVQMAYRKKAETDRRGGFADEASASTESDIDETLQRLVGSLGKTPREVFDALRSQTIDLVLTAHPTQSVRRSLLQKHGRIRDCLTQLCVDGIAENERREIDEALQREASDAIQFFLPSDGVVDDANCNRLESLPPCSLQILAAFRTDEIRRTQPTPQDEMRAGMSYFDDTIWNGVPKFLRRVDTALKNIGIDERLPYDAPLIQFSSWMGGDRDGNPRVTPEVTRDVCLLARMMAANMYFSKMAGLMFELSMWRCNDELRARADELHRQSSRKYAKYYIEFWKQISPREPYRIVLGDVRDKLYNTCERARQILSHGVSSIPEDKTYVNVKQFLEPLELCYRSLCDCGDKLIADGNLLDFMRQVSTFGLSLVKLDIRQESERHTDAMDAITTHLGIGSYREWPEEQRQEWLVSELRGKRPLFGPDLPQSEEVADVLGTFRVIAELPDDSFGAYIISMATAPSDVLAVELLQRECGIKRPLRVVPLFEKLADLQQGPATMELLFSIDWYKQRIGGKQEIMIGYSDSGKDAGRLSAAWQLYKAQEEIVGVAERHGVKLTIFHGRGGTVGRGGGPSHLAILSQPPNTVNGSLRVTVQGEVIEKSFGEENLCFRTLQRFTAATLEHGMNPPVSPKPEWRRLLDDMAAVSTEEYRSIVFQEPRFVEYFRAATPETEYGRMNIGSRPSKRKPGGGIESLRAIPWIFAWTQTRFHLPVWLGFGAAFRHAAGTPGGLATLREMYDEWPFFRVTIDLLEMVFAKGDPGIAALYDKLLVPDDLRPFGEQLRANYAETQSLLLQVAGHKDLLESDPYLRQRLMLRDSYITALNACQAYTLKRIRDGGFRPAARGAPLSKELLGSASTAEGLVKLNPSSEYDPGLEDTLILTMKGIAAGMQNTG

>Do005685.1

MAAKAPMERHQSIDAQLRLLAPGKVSEDDKLVEYDALLVDRFLDILQDLHGPHLREFVQECYELSAEYENDGDEARLGELGSKLTSLPPGDSIVVASSFSHMLNLANLAEEVQIAHRRRIKLKRGDFADEASAPTESDIEETLKRLVSQLGKSREEVFDALKNQTVDLVFTAHPTQSVRRSLLQKHGRIRNCLRQLYAKDITADDKQELDEALQREIQAAFRTDEIRRTPPTPQDEMRAGMSYFHETIWKGVPKFLRRIDTALKNIGIDERLPYNAPLIQFSSWMGGDRDEFWKQVPPNEPYRVILGDVRDKLYYTRERSRHLLTTGISEIPEEATFTNVEQFLEPLELCYRSLCACGDKPIADGSLLDFLRQVSTFGLALVKLDIRQESDRHTDVLDSITTHLGIGSYAEWSEEKRQDWLLSELRGKRPLFGSDLPMTEETADVLGAFHVIAELPADCFGAYVISMATAPSDVLAVELLQRECHVKQPLRVVPLFEKLADLEAAPAAVARLFSIDWYMNRINGKQEVMIGYSDSGKDAGRLSAAWQMYKAQEELIKVAKHYGVKLTMFHGRGGTVGRGGGPTHLAILSQPPDTIHGSLRVTVQGEVIEHSFGEEHLCFRTLQRYTAATLEHGMHPPISPKPEWRALMDEMAVVATKEYRSIVFQEPRFVEYFRSATPETEYGRMNIGSRPSKRKPSGGIESLRAIPWIFAWTQTRFHLPVWLGFGAAIKHIMQKDIRNIHTLKEMYNEWPFFRVTLDLLEMVFAKGDPGIAAVYDKLLVSDDLQSFGEQLRKNYEETKELLLQVAGHKDVLEGDPYLKQRLRLRESYITTLNVCQAYTLKRIRDPSFQVSPQPALSKEFTDESQPAQLVQLNPESEYAPGLEDTLILTMKGIAAGMQNTG
