# Supplementary material for: The draft genome of the C3 panicoid grass species Dichanthelium oligosanthes
Source: Genome Biol. 2016 Oct 28;17:223. doi: 10.1186/s13059-016-1080-3 (PMC5084476; doi:10.1186/s13059-016-1080-3)
Supplement: Additional file 5: — Carbonic anhydrase coding sequences. (DOCX 37 kb) [file 13059_2016_1080_MOESM5_ESM.docx]

***Carbonic Anhydrase* coding sequences.**

>GRMZM2G121878_1

ATGTACACATTGCCCGTCCGCGCCACCACATCCAGCATCGTCGCCAGCCTCGCCACCCCCGCGCCGTCCTCCTCCTCCGGCTCCGGCTCCGGCCGCCCCAGGCCCAGGCTCATCCGGAACGCCCCCGTCTTCGCCGCCCCCGCCACCGTCGTGGGCATGGACCCCACCGTCGAGCGCTTGAAGAGCGGGTTCCAGAAGTTCAAGACCGAGGTCTATGACAAGAAGCCGGAGCTGTTCGAGCCTCTCAAGTCCGGCCAGAGCCCCAGGTACATGGTGTTCGCCTGCTCCGACTCCCGCGTGTGCCCGTCGGTGACACTGGGCCTGCAGCCCGGCGAGGCATTCACCGTCCGCAACATCGCCTCCATGGTCCCACCCTACGACAAGATCAAGTACGCCGGCACCGGGTCCGCCATCGAGTACGCCGTGTGCGCGCTCAAGGTGCAGGTCATCGTGGTCATTGGCCACAGCTGCTGCGGTGGCATCAGGGCGCTCCTCTCCCTCAAGGACGGCGCGCCCGACAACTTCCACTTCGTGGAGGACTGGGTCAGGATCGGCAGCCCTGCCAAGAACAAGGTGAAGAAAGAGCACGCATCGGTGCCGTTCGATGACCAGTGCTCCATCCTGGAGAAGGAGGCCGTGAACGTGTCGCTCCAGAACCTCAAGAGCTACCCCTTCGTCAAGGAAGGGCTGGCCGGCGGGACGCTCAAGCTGGTTGGCGCCCACTACGACTTCGTCAAAGGGCAGTTCGTCACATGGGAGCCT

>GRMZM2G121878_2

CCCCAGGACGCCATCGAGCGCTTGACGAGCGGGTTCCAGCAGTTCAAGGTCAATGTCTATGACAAGAAGCCGGAGCTTTTCGGGCCTCTCAAGTCCGGCCAGGCCCCCAAGTACATGGTGTTCGCTTGCTCCGACTCCCGTGTGTGCCCATCGGTGACCCTGGGCCTGCAGCCCGGCGAGGCCTTCACCGTTCGCAACATAGCCGCCATGGTCCCAGGCTACGACAAGACCAAGTACACCGGCATCGGGTCCGCCATCGAGTACGCTGTGTGCGCTCTCAAGGTGGAGGTCCTCGTGGTCATTGGCCATAGCTGCTGCGGTGGCATCAGGGCGCTCCTCTCCCTCCAGGACGGCGCACCTGACACCTTCCACTTCGTCGAGGACTGGGTTAAGATCGGCTTCATTGCCAAGATGAAGGTAAAGAAAGAGCACGCCTCGGTGCCGTTCGATGACCAGTGCTCCATTCTCGAGAAGGAGGCCGTGAACGTGTCCCTGGAGAACCTCAAGACCTACCCCTTCGTCAAGGAAGGGCTTGCAAATGGGACCCTCAAGCTGATCGGCGCCCACTACGACTTTGTCTCAGGAGAGTTCCTCACATGGAAAAAGTGA

>GRMZM2G348512_CA2

ATGGACGACCCCGTCGAGCGCTTGAAGGACGGGTTCCACAAGTTCAAGACCGAGGTCTATGACAAGAAGCCGGAGCTGTTCGAGCCTCTCAAGGCCGGCCAGGCCCCCAAGTACGTGGTGTTCGCCTGCTCCGACTCCCGCGTGTGCCCGTCGGTGACCCTGGGCCTGCAGCCCGGCGAGGCCTTCACCGTCCGCAACATCGCCGCCATGGTCCCAGCCTACGACAAGACCAAGTACACCGGCATCGGGTCCGCCATCGAGTACGCCGTGTGCGCGCTCAAGGTGGAGGTCCTCGTGGTCATTGGCCACAGCTGCTGCGGTGGCATCAGGGCGCTCCTCTCCCTCCAGGACGGCGCACCTGACAACTTCCACTTCGTTGAGAACTGGGTTAAGATCGGCTTCCCTGCCAAGATTAAGGTGAAGAAAGACCACGCATCGGTGCCGTTCGATGACCAGTGCTCTATTCTAGAGAAGGAGGCCGTGAACCTGTCCCTGGAGAACCTCAAGACCTACCCCTTCGTCAAGGAAGGGCTGGCCAACGGGACGCTCAAGCTGGTCGGCGGCCACTACAACTTCGTGTCAGGGGAGTTCCTCACATGGGACAATAAGCAGCCATCGTGA

>GRMZM2G348512_CA3

ATGGCCGTCGAGCGCTTGAAGACCGGGTTCGAGCAGTTCAAGGCCGACGTCTACGACAAGAAGCCGGAGCTGTTCGAGCCACTCAAGGCGCACCAGTCGCCCAAGTACATGGTGTTCGCCTGCTCCGACTCCCGCGTGTGCCCGTCGGTGACCCTGGGCCTGCACCCCGGCGAGGCCTTTGCCGTCCGCAACATCGCCAGCATGGTGCCGCCCTACGACAAGACCAAGTACGCCGGCGTCGGCTCCGCCATCGAGTACGCCGTCTGCGCCCTCAAGGTGGAGGTCATCGTGGTCATAGGCCACAGCCGCTGCGGCGGGATCAAGGCGCTCCTCTCCCTCGAGGACGGCGCGCCCGACAAGTTCCACTTCGTGGAGGAATGGGTCAGGGTCGGCGCCCCGGCGAAGTCCAAGGTGCTGGCCGACCACGCCTCGGCGCCTTTCGAAGACCAATGCTCCATCCTGGAGAAGGAGGCCGTCAACGTGTCCCTTGAGAACCTCAAGTCCTACCCGTTCGTCAAGGAAGGGCTGGAGAAAGGGACCCTCAAGCTGGTCGGCGGCCATTACGACTTCGTCAATGGAAAGTTCGAGACATGGGAACCCTAA

>Sobic.003G234200_1

ATGTCCGCCTTCCCCGTCCGCGGCTCCACGTCCAGCAGCAGCATCGTCGCCAGCCTCGGCAGCAGCCGCGCCCCCGCCCCCGCGCCGTCCTCCGTCCGCCGGCGCCCCAGCCTCATCAGGAACGCCCCCGTCGTCGCCGTCCCCGCCACCCTCGTGGGCATGGACGCCGTCGAGCGCTTGAAGGTCGGGTTCCAGAAGTTCAAGACCGAGGTCTATGACAAAAAGCCAGAGCTGTTCGAGCCTCTCAAGGCTGGCCAGGCCCCCAAGTACATGGTGTTCGCCTGCTCCGACTCCCGTGTGTGCCCGTCGGTGACCCTGGGCCTGCAGCCCGGCGAGGCCTTCACCGTCCGCAACATCGCCTCCATGGTGCCACCCTACGACAAGACCAAGTACAGCGGCACCGGGTCCGCCATCGAGTACGCCGTGTGCGCCCTCAAGGTGCAGGTGCTCGTCGTCATTGGCCACAGCTGCTGCGGTGGCATCAGGGCGCTCCTCTCCCTCAAGGACGGTGCACCTCACAACTTCCACTTCGTTGAGGACTGGGTCAGGATCGGCTCCCCTGCCAAGGCGAAGGTGCACAAAGAGCATGCCTCAGTGCCGTTCGATGACCAGTGCTCCATCCTGGAGAAGGAGGCCGTGAACGTGTCCCTCGCCAACCTCAAGACTTACCCCTTCGTCAAGGAACGGCTGGCCAAAGGGACCCTCAAGCTGGTCGGCGCCCACTACGACTTCGTGTCCGGGAAGTTCCTCACATGGGAGCCTGTG

>Sobic.003G234200_2

ATGGACGCCGTTGACCGCTTGAAGAGCGGGTTCGAGCAGTTCAAGATCGAGGTCTATGACAAGAAGCCAGAGCTTTTTGAGCCTCTCAAATCTGGCCAGGCCCCCAGGTACATGGTGTTCGCCTGCGCTGACTCCCGTGTGTGCCCGTCGGTGACCCTGGGCCTGATGCCCGGCGAGGCCTTCACCGTCCGCAACATCGCCGCCCTGGTCCCAGGCTACGACAAGACCAAGTACACCGGCATCGGGTCCGCCATCGAGTACGCCGTGTGCGCCCTCAAGGTGCAGGTGCTCGTCGTCATTGGCCATAGCTGCTGCGGTGGCATCAGGGCGCTCCTCTCCCTCCAGGACGGTGCACCTGACAACTTCCACTTTGTTGAGGACTGGGTCAAGATTGGCTTCCCTGCCAAGATGAAGGTGAAGAAAGAGAACGCTTCGGTGCCGTTCGATGACCAATGCTACATTCTAGAGAAGGAGGCCGTGAATGTGTCCTTGGAGAACCTCAAGACCTACCCATTCGTCAAGGAAGGGCTGGCCAACGGTACCTTAAAGCTGGTCGGCGGCCACTATGACTTCGTGAAAGGAGAGTTCCTCACATGGAAAGTCTAG

>Sobic.003G234400

ATGGCGGACGCCGTCGACCGCTTGAAGACCGGGTTCAACAAGTTCAAGACCGAGGTCTATGACAAGAAGCCGGAGCTGTTCGAGCCTCTCAAGGCCGGCCAGTCCCCCAAGTACATGGTGTTTGCGTGCTCCGACTCCCGTGTGTGCCCGTCGGTGACCCTGGGTCTGCAGCCCGGTGAGGCCTTCACCGTCCGCAACATCGCCGCCATGGTCCCAGCCTACGACAAGACCAAGTACACCGGCATTGGGTCTGCCATTGAGTACGCCGTGTGCGCCCTCAAGGTGCAGGTCCTCGTCGTCATTGGCCACAGCTGCTGTGGTGGCATCAGGGCGCTCCTCTCCCTCCAGGATGGCGCACCTGACACCTTCCACTTCGTTGAGGACTGGGTCAGGATCGGCTTCCCTGCCAAGATCAAGGTGAAGAAAGAGCACGCTTCGGTGCCGTTCGATGACCAGTGTTCCATCCTGGAGAAGGAGGCCGTGAACGTGTCCCTCGACAACCTCAAGACGTACCCCTTCGTCAAGGAAGGGCTGGCCAACGGGACCCTCAGGCTGGTCGGCGGCCACTACAACTTCGTGTCAGGGGAGTTCGACACATGGGAAATTAAGAGCTAA

>Sobic.003G234500

ATGGCGGACGCCGTCGAGCGCTTGAAGACCGGGTTCGAGCAGTTCAAGGCCGATGTCTACGACAAGAAGCCGGAGCTGTTCGAGCCACTCAAGGCGCACCAGTCACCCAAGTACATGGTGTTCGCCTGCTCCGACTCCCGCGTGTGCCCGTCGGTGACCCTGGGCCTGCACCCCGGCGAGGCCTTCGCCGTCCGCAACATCGCCAGCATGGTGCCACCCTACGACAAGACCAAGTACGCCGGCGTCGGGTCCGCCATCGAGTACGCCGTGTGCGCCCTCAAGGTGGAGGTCATCGTCGTCATCGGCCACAGCCGCTGCGGCGGGATCAAGGCGCTCCTCTCCCTCGAGGACGGCGCGCCCGACAAGTTCCACTTCGTGGAGGAATGGGTCAGGATCGGCGGTCCGGCCAAGACTAAGGTGAAGACCGACCACGCCTCGGTGCCGTTCGAAGACCAATGCTCCATCCTCGAGAAGGAGGCCGTGAACGTGTCCCTTGAGAACCTCAAGACGTACCCGTTCGTCAAGGAAGGGGTGGAGAAAGGGACCCTCAAGCTGGTCGGCGGCCACTACGACTTCGTCAATGGAAAGTTCGAGACATGGGAGCCCTAA

>Sobic.003G234600

ATGGGGGGCTGCTGCTGCGGCTGCGGCTGCGGCTGCGGCTGCTTCCTCGACCGAAAGCCTCCGCGGGAAAACCCGATGGCGGTGCATCGGGAACAGCCCATCCGGCGCGGGTCGAGCGCTGCCGACTACCCCTCCCCAGCACATATGGTCACGTACAGCGAGGGGCTGGGTGCTGCGGAGAGATTGAACGCTGGGTTCCGGACGTTTAAAAAGAACGTCTACGATCAGAACCCAAAGTTGTTCGGCAAGCTCAAGTCTGCCCAGTCTCCGAAGTACATGGTGTTTGCATGCTCTGACTCGCGTGTTTGTCCATCAGTGACCCTTGACCTAAAGCCAGGAGAAGCCTTTACTGTCCGTAATATTGCCAGCCTAGTCCCAACCTACAAGCAGAATGTGCACTGCAATATCGGTTCAGCCATCGAGTATGCTGTGACCATCCTCAAGGTTGAGTGTATTGTGGTTATTGGTCACAGCTGCTGTGGTGGAATCAGGGCACTCCTCTCTTTGAAGGAAGATAGACCACACACCTACCACTTCATTGAAAACTGGGTTAAGATCGGTCTGGATATCAAGAAGAAGGTGGAGAGAATACATGCCTTTCTGCCTTTTGATGAACAATGCACCAAGTTGGAAATTGAGGCCGTCAATTTGTCCCTTAGAAACCTGAAGACCTATCCATTTGTCATGGATAGATTGGCCAAGGGAACACTCAAACTCATTGGTGCTCGCTACGACTTTGTTTGTGGCACCTTCCAGACTTGGAATGCCTGA

>Si003882m

ATGTCTACTTGCTTCGCTGGGTTGAGCATCGTCGCAAGCCTCGGCACCCCCAAGCCGTCCTCCGCCTCCGCACGCCCCAGGCTCATCAGGAACGCGCCCGTCTTCGCCGCCGCCACCGCCGTCGTGGGCATGGACGCCGTCGAGCGCTTGAAGAGCGGGTTTGACAAGTTCAAGACCGATGTCTATGACAAGAAGCCGGAGCTGTTCGAGCCGCTCAAGGAGGGCCAGGCACCCAGGTACATGGTGTTTGCCTGCTCCGACTCCCGTTGCTGCCCGTCGGTGACCCTGGGCCTGCAGCCCGGCGAGGCCTTCACCGTCCGCAACATCGCCGCCATGGTCCCACCTTACGACAAGACCAAGTACACCGGCATTGGGTCCGCCATCGAGTACGCCGTCTGCGCCCTCAAGGTGGAGGTCCTCACCGTCATCGGCCACAGCCGCTGCGGTGGCATCAAGGCGCTCCTCTCCATGAAGGACGGCGCACCTGACAACTTCCACTTCGTCGAGGACTGGGTCAGGATCGGCTTCCTCGCCAAGAAGAAGGTGCTGACCAACCATGCCTCGGTTCCGTTCGATGACCAGTGCACCCTCTTGGAGAAGGAGGCCGTCAACGTGTCCCTCTACAACCTCCTGACCTACCCCTGGGTGAAGGAAGGTGTGGCCAACGGGACACTCAAGCTGGTCGGCGGCCACTACGACTTCGTCAACGGGGTGTTCTCCGTGTGGGAGAAATAA

>Si002140m

ATGGCGGACGCCGTCGAGCGCTTGAAGACCGGGTTCGAGCAGTTCAAGGCCGATGTCTACGACAAGAAGCCGGAGCTGTTCGAGCCGCTCAAGGCCCACCAGTCCCCCAAGTACATGGTGTTCGCCTGCTCCGACTCCCGCGTGTGCCCGTCGGTGACCCTGGGCCTGCAGCCCGGCGAGGCCTTCGCTGTCCGCAACATCGCCAGCATGGTCCCACCCTACGACAAGACCAAGTACGCCGGCGTCGGGTCCGCCATCGAGTACGCCGTCTGCGCCCTCAAGGTGGAGGTCATCGTCGTCATCGGCCACAGCTGCTGCGGCGGGATCAAGGCGCTCCTCTCGCTCCAGGACGGCGAGGCCGACAAGTTCCACTTCGTCGAGGACTGGGTCAGGATCGGCGCCCCCGCGAAGGCGAAGGTGCAGGCCGACCACGCCTCAGCTGCTTTCGAGGACCAATGCTCCATCTTGGAAAAGGAGGCCGTCAACGTGTCCCTTGAGAACCTCAAGACCTACCCGTTCGTCAAGGAAGGGCTGGAGAAGGGGACCCTCAAGCTGGTCGGCGGCCACTACGACTTCGTCTCCGGCAAGTTCGAGACATGGGAACCCTAA

>Si002669m

ATGGGGGGATGCTGCTGCTGCTTCCTCGCTCACAAGCCGCCGCGGGAGAACCCGATGCACTCGTCCCGGGAGCCGCTCATCTGGAGCGGATCGGGCGCTGCCGGCCACCACCACCACCCGGCTCAGATGGTCACCTACAGCGAGGGGTTGGGTGCTGCGGAGAGATTGAGGGCTGGGTTCAGGACGTTTAAGAGGACTATTTATGATAAGAACCCCATGCTGTTCGGGCCGCTCAAGTCTGCTCAGTCCCCAAAGTACATGGTGTTTGCGTGCTCTGACTCTCGTGTATGCCCATCAGTGATCCTCGACCTGAAACCAGGAGATGCCTTTACTGTCCGTAACATTGCCGGCCTTGTTCCGGCCTACCATCAGAATATGCACTCCAGCGTTGCTTCAGCCATTGAGTTTGCTGTGACCATCCTCAAGGTTAAGTGCATTGTGGTTATTGGTCACAGCTGCTGTGGTGGAATCAGGGAACTCCTCTCTCTGAAGGAAGATAGACCTCAAACCTACCACTTCATTGACAATTGGGTCAAGATCGGTCTGGCTATCAAGAAAAAGGTGGAGCGAGAACATGCCTTATTGTCTTTTGATGACCAATGCACCATGTTGGAAATTGAGGCCGTCAATTTGTCCCTTAGAAACCTGACGACCTACCCATTCGTCAAGGACAAATTGGGCAAGGGAACGCTCAAGCTGATTGGTGCTCGCTACGACTTTGTCCATGGCAGCTTCCAGACGTGGCATGCCTGA

> Do030154

ATGTCGACCTTCGCCGTCCGCGCCTCCGCGTCCAGCATCGTCGCCAGCCTCGGCACCCCCGCGCCGTCCTCCTCCAACAGCCCCGCCTCCGCGCGCCCAAGGCTCATCAGGAACGCGCCCGTCTTCGCCGCCCCTGCCACCGTCGTGGGCATGGACGCCGTCGAGCGCTTGAAGAGCGGGTTCGAGCAGTTCAAGACCGAGGTCTATGACAAGAAGCCGGAGCTGTTCGAGCCGCTCAAGGCCGGCCAGGCCCCCAGGTACATGGTGTTCGCCTGCGCCGACTCCCGTGTGTGCCCGTCGGTGACCCTCGGCCTGCAGCCCGGCGAGGCCTTCACCATCCGCAACATCGCCGCCATGGTCCCACCCTACGACAAGAACCGGTACACCGGCATCGGGTCCGCCATCGAGTACGCCGTCTGCGCCCTCAAGGTGGAGGTCCTCACCGTCATCGGCCACAGCCGCTGCGGTGGCATCAAGGCGCTCCTTTCCCTCCAGGACGGCGCGGCCGACAACTTCCACTTCGTCGAGGACTGGGTCAGGATCGGGTACCTCGCTAAGAAGAAGGTGCAAACCGAGCACGCCTCGGTTCCGTTCGATGAGCAGTGCTCCATCTTGGAGAAGGAGGCCGTGAACGTGTCCCTGGACAACCTCCTGACCTACCCCTGGGTCAAGGAAGGAGTGGCCAACGGGACCCTCAAGCTGGTGGGCGGCCACTACGACTTCGTGGCCGGGGAGTTCCTCACCTGGGAGAAATGA

> Do030155

ATGGCGGACGCCGTCGAGCGCTTGAAGACCGGGTTCGAGCAGTTCAAGGCTAATGTCTTCGACAAGAAGCCGGAGCTGTTCGAGCCGCTCAAGGCCCACCAGTCCCCCAAGTACATGGTGTTCGCCTGCTCCGACTCCCGCGTTTGCCCGTCGGTGACCCTGGGCCTGCAGCCCGGCGAGGCCTTCACCGTCCGCAACATCGCCAGCATGGTCCCACCCTACGACAAGACCAAGTACGCCGGCGTCGGGTCCGCCATCGAGTACGCCGTCTGTGCCCTCAAGGTGGAGGTCATCGTCGTCATCGGCCACAGCCGCTGCGGTGGGATCAAGGCACTCCTCTCGCTCCAGGACGGCGAGCCCGACAAGTTCCACTTCGTCGAGGAATGGGTCAGGATCGGCGCCCCCGCGAAGACGAAGGTGCAGGCCGATCACGCGTCAGTGCCGTTCGAAGACCAATGCTCCATCCTGGAAAAGGAGGCCGTCAACGTTTCCCTTGAGAACCTCAAGTCCTACCCGTTCGTCAAGGAAGGGCTGGAGAAAGGGACCCTCAATATAGTCGGCGGCCACTACGACTTCGTCTCCGGCAAGTTCGAGACATGGGATGCCTAA

> Do001291.1

ATGGGGGGATGCTGCTGCTGCTTCCCCGCGCGCAAGCCGCCGCGGGAGAACCCGATGCACCCGGCTCGGGAGCCGCTCATCCGGCCTGGATCGAGCGCCGCCGGCCACCATCACCCGGCGCAGATGGTCACCTACAGCGAGGGGTTGGGCGCTGCAGAGAGATTGAAGGCTGGGTTCAGGACGTTTAAGAGGACTATCTATGATCAGAACCCAATGCTGTTCGGGCCGCTCAAGTCTGCACAGTCCCCAAAGTACATGGTGTTTGCGTGCTCTGACTCTCGTGTATGCCCATCGGTGACGCTCGACCTGAAACCAGGAGAGGCCTTTACTGTCCGTAATATCGCCAGCCTGGTTCCAGCCTACCATCAGAATATGCACTCCAGCGTTGCGTCGGCCATTGAGTTTGCTGTGACCATCCTCAAGGTTGAGTGCATTGTGGTTATTGGTCACAGCTGCTGTGGTGGAATTAGGGAACTCCTTTCTTTGAAGGAAGATAGCCCTCACACCTACCACTTCATTGACAACTGGGTGAAGATCGGTCTGGCTATCAAGAAGAAGGTGGAGCACGAACATGCCTTATTGTCTTTTGAGGACCAATGCACCATGTTGGAAATTGAGGCCGTCAATTTGTCCCTTAGAAACCTGAAGACCTACCCATTTGTCAAGGACAAATTGACCAAGGGGACGCTCAAGTTGATCGGTGCTCGCTATGACTTTGTCCATGGCAGCTTCAATACGTGGCATGCCTGA

>Pavir.J05107

ATGAGTAAGTCGTCCGCACTCCACTCGAATACTCGATGCAGCACCCGCCGGCGTCCGATTCCAGTTCACACGGCGCCTGATTCCTTTGGGGAATTTTCGGGAGCTCCGACTGACACTGGATTTCCTCTCTTCTGCAGCAAGAAGCCGGAGCTGTTCGAGCCGCTCAAGGCCGGCCAATCCCCCAGGTACATGGTTTTCGCCTGCGCCGACTCCCGCTGCTGCCCGTCGGTGACCCTGAGCCTGCAGCCCGGCGAGGCCTTCACCATGCGCAACATCGCCGCCATGGTCCCGCCCTACGACAAGAACAAGTACGCCGGCATCGGGGCCGCCATCGAGTACGCCGTCTGCGCGCTCAAGGTGGAGGTCCTCACCGTCATTGGCCACAGCCGCTGCGGTGGCATCAAGGCGCTCCTCTCCCTCCAGGACGGCGCAGCCGACACCTTCCACTTCGTCGAGGACTGGGTCAGGATCGGCTTCCAGGCCAAGAAGAAGGTGCTGAAAGAGCACCCCCATGCTCCGTTCGATGACCAGTGCTCCATCTTGGAGAAGGAGGCCGTGAACGTGTCCCTCTACAACCTCTTGACCTACCCCTGGGTCAAGGAAGGTGTGGAGAACGGGACCCTCAAGCTGGTCGGCGCACGCTACGACTTCGTGAACGGTGTGTTCGACACATGGGTGAAATAA

>Pavir.J05108

ATGGCGGATGCCGTCGAGCGCTTGAAGACCGGGTTCGAGCAGTTCAAGGCCGATGTCTACGACAAGAAGCCGGAGGTGTTCGAGCCGCTCAAGGCCCACCAGTCCCCCAAGTACATGGTGTTCGCCTGCTCCGACTCCCGCGTGTGCCCGTCGGTGACCCTGGGCCTGCAGCCCGGCGAGGCCTTCGCCGTCCGCAACATCGCCAGCATGGTCCCACCCTACGACAAGACCCGGTACGCCGGCGTCGGGTCCGCCATCGAGTACGCCGTCTGCGCGCTCAAGGTGGAGGTCATCGTGGTCATCGGCCACAGCCGCTGCGGCGGGATCAAGGCGCTCCTCTCGCTCAAGGATGGCGAGCCCGACAAGTTCCACTTCGTCGAGGATTGGGTCAGGATCGGCGGCCCCGCGAAGACGAAGGTGCTGGCCGACCACGCCTCAGCCCCCTTCGAAGATCAATGCTCCGCCTTGGAAAAGGAAGCCGTCAACGTGTCCCTCGAGAACCTCAAGACCTACCCGTTCGTCAAGGAAGGGCTGGAGAAGGGGACCCTCAAGCTGGTCGGCGGTCACTACGACTTCGTCTCCGGCAAGTTCGAGACATGGGATCCATGA

>Pavir.J05109

ATGGGGGGATGCTGGTGCTGCTTCCTCGCCCACAAGCCGCCGCGGGAGAACCCGATGAGCCCGGCCCGGGAGCGGCTCATCGGGGGCGGATCGAGCGCTGCCGGCCACCACCACCCGCCCCTGATGGTCACCTACAGCGAGGGGTTGGGCGCTGCGGAGAGATTGAGGGCTGGGTTCAGGACGTTTAAGAGGACCATCTATGATAAAAACCCCATGCTGTTCGGGCCGCTCAAGTCTGCCCAGTCCCCGAAGTACATGGTGTTTGCGTGCTCTGACTCTCGTGTATGCCCATCAGTAACCCTCGACCTGAAACCAGGAGAGGCCTTTACTGTCCGTAACATTGCCAGCCTGGTTCCGGCCTACCACCAGAATATGCACTCTAGCGTTGCGTCGGCCATTGAGTTTGCTGTTACCATTCTCAAGGTTAAGTGCATTGTGGTTATTGGTCACAGCTGCTGCGGTGGAATCAGGGAACTCCTCTCTCTGCAGGAAGATAGACCTCACACCTACCATTTCATTGACAATTGGGTTAAGATCGGTCTGGCTATCAAGAAGAAGGTGGAGCGAGAGCATGCCTTATTGTCTTTTGATGACAAATGCACCATGTTGGAAATTGAGGCTGTCAATTTATCCCTTAGAAATCTGAAGACCTACCCATTTGTCAAGGACAAATTAGCCAACGGATCGCTCAAGTTGATTGGTGCTCGCTATGACTTTGTCCATGGCAGCTTCCAGACTTGGCATGCCTGA

>Bradi2g44856

ATGTCGACCGCCGCGGCCAACTGGTGCTACGCAACCGTCGCGCCCCGCGCTAAGAGCGTCGTCGTCGCCAGCCTCGGCACCCCGGCCCCGTCCTCTTCCGGCAGCTTCCGGCCCAGGCTCATCAGGAACGCCCCCGTCCAGGCCGCGCCCGTCGCGCCCGCATTGATGGACGCCGCCGTGGAGCGCCTCAAGACCGGGTTCGAGAAGTTCAAGACCGAGGTCTACGACAAGAAGCCGGATGTCTTCGAGCCGCTCAAGGCCGGCCAGGCCCCCAAGTACATGGTGTTCGCCTGCGCCGACTCACGTGTGTGCCCGTCGGTGACCCTGGGCCTGGAGCCCGGTGAGGCCTTCACCGTCCGCAACATCGCCAACATGGTCCCGTCCTACTGCAAGAACAAGTACGCCGGTGTTGGGTCGGCCATCGAGTACGCCGTGTGTGCCCTCAAGGTTGAGGTCATCGTGGTGATTGGCCACAGCCGCTGCGGTGGAATCAAGGCACTCCTCTCGCTCAAGGATGGTGCAGATGACAGCTTCCACTTCGTCGAGGACTGGGTCAGGATCGGGTTCCCGGCCAAGAAGAAGGTGCAGACCGAGTGCGCCTCCATGCCTTTCGATGACCAATGCGCCGTCTTGGAAAAGGAGGCCGTGAACGTGTCCCTCGAGAACCTCAAGACCTACCCGTTCGTCAAGGAAGGCGTCGCCAACGGAACCCTCAAGCTCGTGGGCGGCCACTACGACTTCGTCTCCGGCAAGTTCGACACATGGGAGCTCTAA

>Bradi2g44870

ATGGGTGGCTGCTGCTGCTGCTGCTTCCCCGCCTCCGCTAAACCCAGGAGAGAGAACCCAATGCACCCGGCCACGGAGTCACTCATCCAGCGGAATCCGGATCACACGGTTCCACATCATCATCCGCCCCAGGAGGTCAAAAACACTGGCAAGGGCACGAAGGCTGCCGTACGGTTGAAGACCGGGTTCGAGCGTTTTAAGACGACTATCTACGACAAGAATCTGAAGCTGTTTGAGCCGCTCAAGACCAGCCAGACCCCCAAGTACATGGTGTTCGCATGCTCTGACTCGCGCGTGTGCCCAACATTAACCCTCGGCCTGCAGCCAGGAGAGGCGTTCACTGTCCGAAATATTGCGGGCATGGTTCCTGCCTACCAAGAGAAAAGGCACTGCAGCATTGGGTCAGCCATTGAGTTTGCCGTGGTTGTCCTCAAGGTTGAGTGCATTGTTGTGATGGGTCATAGCCGCTGTGCTGGAATCAAGGAACTCCTCTCTCTGAAAGCAGACAGACCTCACGCCTATCACTTCATTGAGGATTGGGTCAAGGTCGGCATGAACGCCAAGAGGAAGGTCCTGAGAGAAAACCGGTTGTTGCCTTTCGACGACCAATGCACCGTGCTGGAAAAGGAGGCCGTCAATGAGTCCCTTGCAAATCTCAAATCCTACCCGTTCGTCAAGGATAGATTGCGCAAGGGCACACTGAGTCTGCTTGGTGCCCGCTACGACTTCGTGAACGTCAGCTTGGAGACGTGGAATCCCTGA

>Os01g45274

ATGTCGACCGCCGCCGCCGCCGCCGCTGCCCAGAGCTGGTGCTTCGCCACTGTCACCCCGCGCTCCCGCGCCACAGTCGTCGCCAGCCTCGCCTCCCCATCACCGTCCTCCTCCTCCTCCTCCTCCAACAGCAGCAACCTCCCGGCCCCCTTCCGCCCCCGCCTCATCCGCAACACCCCCGTCTTCGCCGCCCCCGTCGCCCCCGCCGCGATGGACGCCGCCGTCGACCGCCTCAAGGATGGGTTCGCCAAGTTCAAGACCGAGTTCTATGACAAGAAGCCGGAGCTCTTCGAGCCGCTCAAGGCCGGCCAGGCACCCAAGTACATGGTGTTCTCGTGCGCCGACTCTCGCGTGTGCCCGTCGGTGACCATGGGCCTGGAGCCCGGCGAGGCCTTCACCGTCCGCAACATCGCCAACATGGTCCCAGCTTACTGCAAGATCAAGCACGCTGGCGTCGGGTCGGCCATCGAGTACGCCGTCTGCGCCCTCAAGGTCGAACTCATCGTGGTGATTGGCCACAGCCGCTGCGGTGGAATCAAGGCCCTCCTCTCACTCAAGGATGGAGCACCAGACTCCTTCCACTTCGTCGAGGACTGGGTCAGGACCGGTTTCCCCGCCAAGAAGAAGGTTCAGACCGAGCACGCCTCGCTGCCTTTCGATGACCAATGCGCCATCTTGGAGAAGGAGGCCGTGAACCAATCCCTGGAGAACCTCAAGACCTACCCGTTCGTCAAGGAGGGGATCGCCAACGGCACCCTCAAGCTCGTCGGCGGCCACTACGACTTCGTCTCCGGCAACTTGGACTTATGGGAGCCCTAA

>Os01g45290

ATGGGTGCCTGCTGCTGCTGCTTCCCGGTCTACAAACCCGCGAGGGAGAACCCGATGCGGTCGACCAGAGAGTCACTCATCCAGCACAAGCCGAGGCCTACCACGCCGTACCATCCACCACCACCCCCGTTTATCACCTACACTGACAAGGGCATGAACGCTGTCGAGCGCTTCAAGACTGGGTTCGAGAATTTCAGGAATACTATATACGACAAGAGGCCGGAGCTGTTTGAGCGGCTCAAGACTGGCCAGTCACCAAAGTACATGGTTTTCTCGTGTGCTGACTCACGTGTGTGCCCAACACTGACCTTCGGTCTACAACCTGGTGAGGCCTTCACTGTCCGTAATATTGCCAGCATGGTTCCAGCCTATGACAAGAGAGGGCAGTGCAGCATTGGGTCAGCCATTGAGTATGCTGTGGTTGTCCTCAAGGTTGAATGTATCATTGTGATTGGTCACAGCTGCTGTGGTGGAATCAAGGAACTCCTCTCTCTGAAGGAAGATAGACCTAACACCTTCCACTTCGTTGATGACTGGGTCAAGATTGGTCTGGCTGCGAAGAAGAAGGTCGAGAGAGAAAACATGTTACTGCCTTTTGACGACCAATGCACCGTGCTGGAAAAGGTATTATTGCTTGGAACAACATTATATTATTTCTAG

>Hv_AK364013.1

ATGTCGACCGCCGCGGCTAACTGGTGCTACGCAACCGTCGCGCCCCGTGCCCGCTCCTCCACCATCGCCGCCAGCCTCGGCACCCCCGCGCCCTCCTCCTCCGCCTCCTTCCGCCCCAAGCTCATCAGGACCACCCCCGTCCAGGCCGCGCCCGTCGCACCTGCATTGATGGACGCCGCCGTGGAGCGCCTCAAGACCGGGTTCGAGAAGTTCAAGACCGAGGTCTACGACAAGAAGCCCGATTTCTTCGAGCCGCTCAAGGCCGGCCAGGCGCCCAAGTACATGGTGTTCGCGTGCGCCGACTCGCGTGTGTGCCCGTCGGTCACCCTGGGCCTTGAGCCCGGTGAGGCCTTCACCATCCGCAACATCGCCAACATGGTCCCGGCCTACTGCAAGAACAAGTACGCCGGTGTTGGATCGGCCATCGAATACGCCGTCTGCGCGCTCAAGGTTGAGGTCATCGTGGTGATTGGCCACAGCCGCTGCGGTGGAATCAAGGCTCTGCTCTCGCTCAAGGATGGCGCAGACGACTCCTTCCACTTCGTTGAGGACTGGGTCAGGATCGGGTTCCCGGCCAAGAAGAAGGTGCAGACTGAGTGCGCCTCCATGCCTTTCGATGACCAGTGCACCGTCCTGGAGAAGGAGGCCGTCAACGTGTCCCTCCAGAACCTCTTGACCTACCCGTTCGTCAAGGAGGGTGTGACCAACGGAACCCTCAAGCTCGTCGGCGGCCACTACGACTTCGTCTCCGGCAAGTTCGAAACATGGGAGCAGTAA

>Ta_AK334385.1

ATGTCGACCGCCGCGGCTAACTGGTGCTACGCAACCGTCGCGCCCCGTGCCCGCTCCTCCACCATCGTCGCCAGCCTCGGCACCCCCGCGCCCTCCAACTCCTCCTCCTTCCGCCCCAAGCTCATCAGGAACACCCCCGTCCAGGCCGCGCCCGTCGCACCCGCATTGATGGACGCCGCCGTGGAGCGCCTCAAGACCGGGTTCGAGAAGTTCAAGACCGAGGTCTACGACAAGAAGCCCGATTTCTTCGAGCCCCTCAAGGCCGGCCAGGCGCCCAAGTACATGGTGTTCGCGTGCGCTGACTCCCGTGTGTGCCCCTCGGTCACCCTGGGCCTGGAGCCCGGCGAGGCCTTCACCATCCGCAACATCGCCAACATGGTCCCGTCCTACTGCAAGAACAAGTACGCTGGTGTTGGATCGGGCATCGAATACGCCGTCTGTGCTCTCAAGGTTGAGGTCATCGTGGTGATTGGCCACAGCCGCTGCGGTGGAATCAAGGCTCTCCTCTCACTCAAGGATGGCGCAGACGACTCCTTCCACTTCGTCGAGGACTGGGTCAGGATCGGCTTCCCGGCCAAGAAGAAGGTGCAGACTGAGTGCGCCTCAATGCCTTTCGATGACCAGTGCACCGTCCTCGAAAAGGAGGCCGTGAACGTGTCCCTCCAGAACCTCTTGACCTACCCGTTCGTCAAGGAGGGTGTGTCCAACGGAACCCTCAAGCTCGTTGGCGGCCACTACGACTTCGTCTCCGGCAAGTTCGAGACATGGGAGCAGTAA

>ITC1587_Bchr9_T25640

ATGGAGCCATTGGAGCAGGTCCAGTCCGGGTTTGTGCAGTTCAAGGAGGAGGTCTATGAGAAGAAGTCGGATTTGTTTGCTGAACTCAAAGAGGGTCAAAGCCCCAAGTTCATGGTGTTCGCATGCGCCGACTCCCGTGTGTGCCCGTCGGTGGTGCTCAACTTCCAGCCCGGTGAAGCCTTCACCATCCGCAACATAGCCAACATGGTCCCTCCCTATGACCAGGTGAAGTATGCTGGTGTCGGAGCGGCCATCGAGTATGCTGTGCTCCATCTCAAGGTGCAGAACATAATGGTCATCGGCCACAGCCGCTGTGGTGGCATCAAGGGACTCATGTCCATCAAGGACGATGGCACCACCAGCACTGACTTCATAGAGGATTGGGTGAAGATTTGCCTACCAGCAAGAGACAAGGTGAAGGCTGAGCACACAGCCTTGTCTTTTGAGGAGCAGTGCACCAAGTGTGAAAAGGAGGCTGTAAATGTGTCCCTGAAAAACCTGAAGACCTACCCCTTCGTCAAAGAAGCTGTAGAGAAAAAATCATTGAAGTTAATTGGAGCGCACTATGACTTTGTCAATGGCATCTTTGAGACATGGGAGGACTAA
